# Development and content validity of the Experienced Patient‐Centeredness Questionnaire (EPAT)—A best practice example for generating patient‐reported measures from qualitative data

**DOI:** 10.1111/hex.13494

**Published:** 2022-04-21

**Authors:** Eva Christalle, Stefan Zeh, Pola Hahlweg, Levente Kriston, Martin Härter, Jördis Zill, Isabelle Scholl

**Affiliations:** ^1^ Department of Medical Psychology University Medical Center Hamburg‐Eppendorf Hamburg Germany

**Keywords:** mixed methods, patient‐centeredness, patient‐reported experience measure, qualitative data, questionnaire development

## Abstract

**Introduction:**

To effectively foster patient‐centeredness (PC), it is crucial to measure its implementation. So far, there is no German measure to assess PC comprehensively. The aim of this study is to develop and select items for the Experienced Patient‐Centeredness (EPAT) Questionnaire, a patient‐reported experience measure (PREM). The EPAT intends to assess PC from the perspective of adult patients treated for different chronic diseases in inpatient and outpatient settings in Germany. Furthermore, we aim at providing a best‐practice example for developing PREMs from qualitative data.

**Methods:**

The development process comprised a three‐phase mixed‐method design: (1) preparation, (2) item generation and (3) item selection and testing of content validity. We generated items using qualitative content analysis based on information from focus groups, key informant interviews and literature search. We selected items using relevance rating and cognitive interviews. Participants were patients from four chronic disease groups (cancer, cardiovascular disease, mental disorder, musculoskeletal disorder) and healthcare experts (e.g., clinicians, researchers, patient representatives).

**Results:**

We conducted six focus groups with a total of 40 patients, key informant interviews with 10 experts and identified 48 PREMs from international literature. After team discussion, we reached a preliminary pool of 152 items. We conducted a relevance rating with 32 experts and 34 cognitive interviews with 21 patients. We selected 125 items assessing 16 dimensions of PC and showed high relevance and comprehensibility.

**Conclusions:**

The EPAT questionnaire is currently undergoing psychometric testing. The transparent step‐by‐step report provides a best practice example that other researchers may consider for developing PREMs. Integrating literature and experts with a strong focus on patient feedback ensured good content validity. The EPAT questionnaire will be helpful in assessing PC in routine clinical practice in inpatient and outpatient settings for research and quality improvement.

**Patient or Public Contribution:**

Patients were not involved as active members of the research team. While developing the funding proposal, we informally reached out to several patient organizations who all gave us positive feedback on the study aims, thereby confirming their relevance. Those patient organizations endorsed the funding proposal with formal letters of support and supported recruitment by disseminating advertisements for study participation.

## 1 INTRODUCTION

During the last decades, the importance of patient‐centeredness (PC) in healthcare and its positive effects on outcomes has been increasingly discussed in the research.^1^, ^2^, ^3^ PC has been incorporated in healthcare agendas and laws by international organizations^4^, ^5^ and national initiatives.^6^ As there was inconsistent use of the term PC, Scholl et al.^7^ integrated different descriptions of PC into a comprehensive framework. The resulting integrative model of PC included 15 dimensions, which were grouped into principles, enablers and activities.^7^ Later, a 16th dimension, patient safety, was added based on a Delphi study with patients.^8^

To effectively foster PC, it is crucial to first assess its status quo. By measuring the degree of implementation of PC, it is possible to identify areas for improvement and evaluate interventions aimed to increase PC.^9^ As PC focuses on patients' experiences, it is highly relevant to assess PC from their perspective. Furthermore, improvement of patient experiences is part of the triple and quadruple aim for enhancing the healthcare system.^10^, ^11^ Patient‐reported experience measures (PREMs) can be used to assess patients' experiences by asking about specific processes and actions in healthcare delivery.^12^ They are different from satisfaction measures, which encompass a more subjective judgement based on individual expectations.^12^ PREMs can be used for quality improvement, benchmarking as well as research and evaluation of interventions.^12^, ^13^

There are several instruments that measure different dimensions of PC.^14^, ^15^, ^16^, ^17^, ^18^, ^19^ To our knowledge there is no German PREM that assesses all 16 dimensions of a PC. Neither did Bull et al.^13^ identify any PREMs in the German language nor any PREM reflecting all dimensions from the integrative model of PC in their systematic review.

We have identified a range of guidelines for developing patient‐reported measures.^20^, ^21^, ^22^ Those guidelines agree on the importance of including the target group (i.e., patients in general) in the item development process as well as using qualitative techniques by conducting focus groups (FGs) or interviews with the said target group. Yet, there is a lack of guidelines on the exact process of deriving items from qualitative data.

Therefore, the aim of this paper was twofold. First, we aimed to report the item development and selection process of the Experienced Patient‐Centeredness (EPAT) questionnaire, a PREM to assess PC from the patients' perspective. Second, we aimed to provide a best‐practice example for developing patient‐reported measures from qualitative data.

## 2 METHODS

### 2.1 Study design

The study was conducted as part of the ASPIRED project (Assessment of Patient‐Centeredness through PREMs), a mixed‐methods study to develop and psychometrically test a measure to assess PC from the patients' perspective.^23^ The development process of the measure consisted of three main phases: (1) preparation, (2) item generation and (3) item selection and testing of content validity, which were based on the guidelines and recommendations for developing patient‐reported measures.^20^, ^21^, ^22^ The Consolidated criteria for reporting qualitative research (COREQ) can be found in Appendix S1.

**Figure 1:**
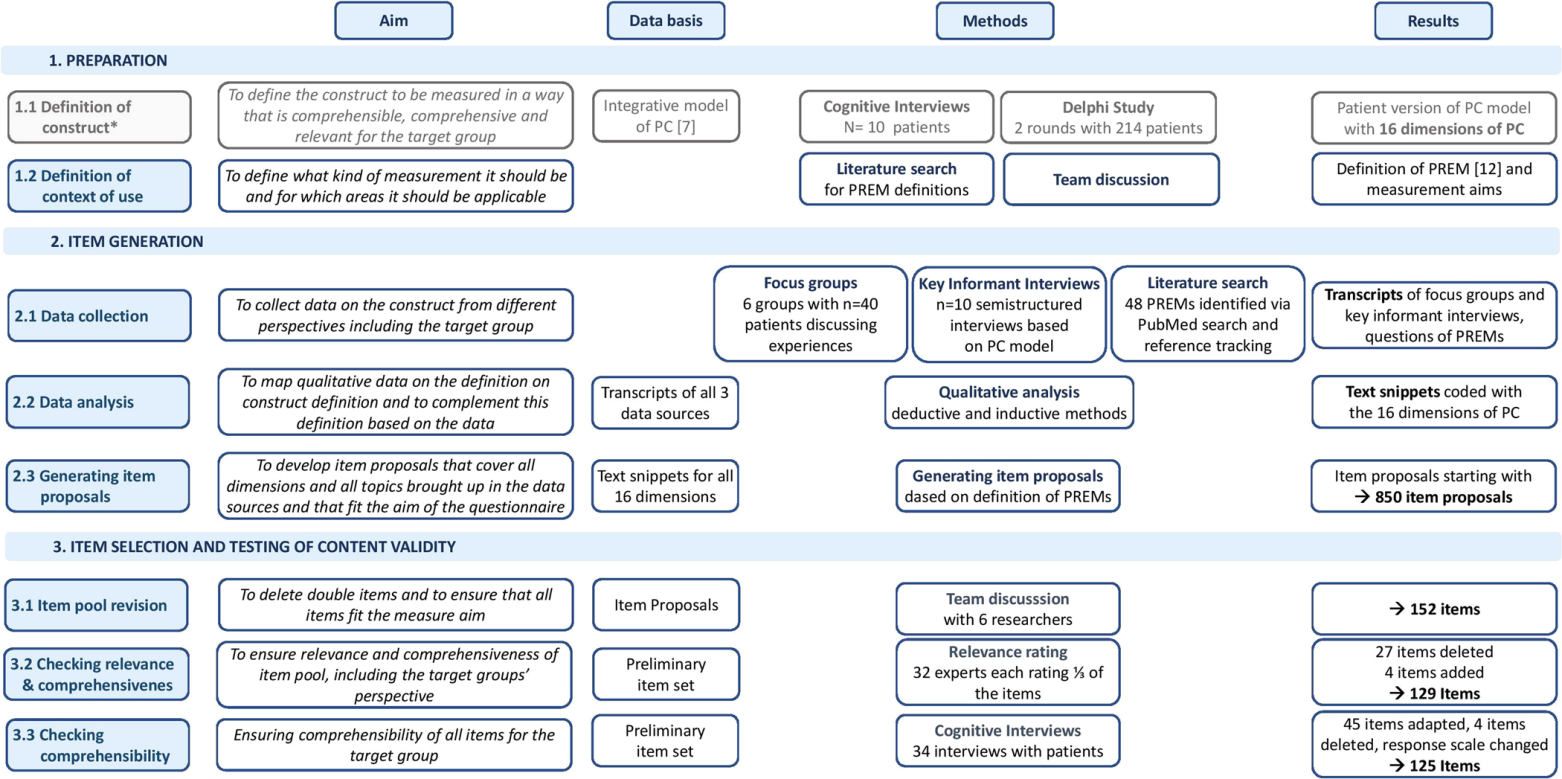
Overview of methods and results of the development of the EPAT questionnaire. *Step 1.1 is not reported in detail as it is already published by Zeh et al. [8]. EPAT, Experienced Patient‐Centeredness; PC, patient‐centeredness; PREM, patient‐reported experience measure

An overview of the study design and methods is given in Figure 1.

### 2.2 Sample

For cognitive interviews and FGs, patients were recruited through community‐based strategies, that is, postings in supermarkets and cooperating self‐help groups and patient organizations. As PC is particularly important for patients with chronic diseases, we focussed on four groups of chronic diseases: cancer, cardiovascular diseases, mental disorders or musculoskeletal disorders. We chose these four groups as they are common and show a high diversity in their course of the disease between and within the disease groups (e.g., constant severity in high blood pressure, episodic courses in depression or remission for long periods in cancer) as well as in their treatments (e.g., surgery for heart diseases, chemotherapy for cancer, psychotherapy for mental disorders). Due to this diversity, we expect that the questionnaire will be generalizable to other diseases. We used a convenience sample including all patients who wanted to participate, self‐reported inclusion criteria were: age (18 years or older) and diagnosis (at least one disease of the four disease groups). All participants gave written consent.

Experts were selected as a convenience sample from the professional network of the research team, for example, collaboration partners, conference contacts, other research projects on PC or by recommendation from experts. The sample included clinicians, healthcare researchers, patient representatives, hospital quality managers and representatives of sickness funds. Experts were contacted via e‐mail or phone and invited to participate in the key informant interviews and/or relevance rating.

### 2.3 Step 1: Preparation

To develop items for a measure, the first step is to define the construct that should be measured and the context of use.^20^, ^24^, ^25^

#### 2.3.1 Step 1.1: Definition of construct

The integrative model of PC was used as a construct for item development.^7^, ^8^ It characterizes PC as follows: Patients have good access to continuous and interdisciplinary care. Healthcare professionals (HCPs) consider the patients as unique persons taking on a biopsychosocial perspective. They establish a good relationship and treat the patients with empathy and respect. The HCPs communicate well and integrate medical and nonmedical care. Patients get tailored information, are empowered to take care of their own health and are involved in treatment decisions. They receive safe care as well as physical and emotional support. For more details on the model and the methods, see Zeh et al.^8^

#### 2.3.2 Step 1.2: Definition of context of use

We searched for definitions of the term PREM and selected the following by Beattie et al.^12^: ‘The emphasis is on asking patients whether or not, or how often, they have experienced certain care processes’.^12^ In our understanding, the term ‘care processes’ encompasses all kinds of behaviours by the HCPs in line with the definition by Bull et al.^13^ that PREMs ‘are tools that capture “what” happened during an episode of care, and “how” it happened from the perspective of the patient’. Further, we decided that the measure should be (1) usable for all four chronic disease groups (i.e., cancer, cardiovascular disease, mental disorder, musculoskeletal disorder), (2) suitable for inpatient and outpatient healthcare delivery for adult patients and (3) applicable for research (e.g., evaluation of interventions) and routine clinical practice (e.g., for quality improvement). As the measure should be used to provide feedback to outpatient and inpatient centres, we focused on the interaction between patients and HCPs and excluded the system level (e.g., interaction with health insurances, conditions due to health policy regulations).

### 2.4 Step 2: Item generation

#### 2.4.1 Step 2.1: Data collection

A combination of deductive (based on literature and existing measures) and inductive methods (generation of items from the responses of individuals) is recommended for developing items for patient‐reported measures.^20^ Therefore, we used three different sources: (1) a *literature search* to identify existing PREMs on PC, (2) *FGs* with patients and (3) *key informant interviews* with experts.

(1) The *literature search* was performed in PubMed with the following search strategy: PREM[Title/Abstract] OR (patient‐reported experience measures[Title/Abstract]) OR (patient‐reported experience measure[Title/Abstract]). No limitations were applied. All references resulting from this search were screened by one researcher (E. C.) and all measures identified were included. Furthermore, references to articles that were included after the first screening and references of a review on PREMs for hospitals^12^ were screened for further measures.

(2) In the *FGs*, we shortly introduced the project, and that our aim was to develop a questionnaire assessing PC. We asked patients to reflect on their own healthcare experiences and to write down three notes to each question: ‘What was good in your healthcare?’ and ‘What should have been done differently in your healthcare?’. Notes were collected and discussed in the group. To focus on specific processes, we asked participants to describe which specific actions the HCPs showed or should have shown in their opinion. FGs had a duration of 120 min (including a break of 15 min).

Focus groups (FGs) were conducted in German at the Department of Medical Psychology of the University Medical Center Hamburg‐Eppendorf (UKE). The FGs were moderated by S. Z. with support from E. C. In addition, I. S. participated in the first group and J. Z. in the last as additional moderators. J. Z. and I. S. (both female) are psychologists, licensed psychotherapists and postdoctoral senior researchers with extensive experience in qualitative research methods. E. C. (female) and S. Z. (male) are junior researchers and PhD candidates. They had training in qualitative research methods and experience with conducting semi‐structured interviews. All had several years of experience as researchers in the field of PC. FG participants did not know the researchers before. All researchers introduced themselves, their professional background and personal motivation as well as the aim of the study.

(3) We conducted semi‐structured *key informant interviews* with a duration of 30–45 min. All key informant interviews were conducted by S. Z. without the presence of third persons. They took place either at the department, at the workplace of the participant or via telephone. We instructed participants to read the model of PC^8^ step by step and describe for each dimension what specific actions by the HCPs are needed to fulfil the respective dimension. Further, participants were to suggest questions that could be used to assess each dimension in a questionnaire.

The guides for the FGs and key informant interviews were designed in German by S. Z. and E. C. with feedback from J. Z. or I. S. They were piloted with student research assistants and research interns. The English translations can be found in Appendix S2 (FGs) and Appendix S3 (key informant interviews).

#### 2.4.2 Step 2.2: Data analysis

FGs and key informant interviews were audio‐recorded, transcribed, and anonymized. No field notes were taken. All analyses were carried out using the transcripts. Neither transcripts nor findings were returned to participants for feedback and no repeat interviews were carried out.

All sources (identified measures and said transcripts) were imported into MaxQDA,^26^ a software specifically designed for qualitative analysis. For analysis of FGs, key informant interviews and identified measures, we used qualitative content analysis based on Mayring.^27^, ^28^ First, we generated a coding system based on the model of PC^8^ (deductive approach). This included 16 codes corresponding to each of the 16 dimensions of the PC. Further, we created subcodes based on the description of each dimension in the model. We further created new codes whenever new topics emerged from the data (inductive approach).

We analysed all data sources by two of three coders (E. C., J. Z. or S. Z.). The first FG as well as the first key informant interview were analysed independently by two researchers (E. C. and S. Z., and J. Z. and S. Z., respectively). All further data sources were coded by one researcher and a second researcher checked the codings and noted cases of disagreement. Then, the two researchers discussed these codings to reach consent. We discussed the codings of every FG/interview/batch of measures before coding the next one to allow for the development of our coding system while ensuring consent on the usage and meaning of every code. No major or minor themes were identified as this is not part of qualitative content analysis based on Mayring and we did not aim to prioritize themes.^27^, ^28^

#### 2.4.3 Step 2.3: Generating items

For each dimension, the text fragments that had been marked by the respective codes and subcodes were collected in one document. Then, one researcher (E. C., J. Z. or S. Z.) used MaxQDA^26^ to develop items based on the text fragments and a second researcher complemented the items. To focus on specific actions of HCPs, we started items with ‘The HCPs did/have/showed…’ whenever possible. The result of this step was a pool of items for each dimension.

### 2.5 Step 3: Item selection and testing of content validity

In the next steps, items were selected. During this process, we checked the item pool for content validity, that is whether the items are ‘relevant, comprehensive and comprehensible with respect to the construct of interest and target population’.^29^

#### 2.5.1 Step 3.1: Item pool revision

As text fragments were often coded with more than one dimension, they were also included more than once in the generation of items. This led to many double items. First, two researchers (E. C. and S. Z.) revised the item pool by sorting the items and merging similar items. Second, iterative group discussions with other researchers followed (P. H., L. K., I. S., J. Z.). These included (1) ensuring that all items were sorted into the best fitting dimension according to the model of PC, (2) deleting all items that did not assess aspects of PC or did not match the context of use, (3) further merging of similar items to eliminate duplicates and (4) refining the wording of the items.

#### 2.5.2 Step 3.2: Checking relevance and comprehensiveness

We conducted a relevance rating with experts (clinicians, healthcare researchers, patient representatives and hospital quality managers) using an online survey via LimeSurvey.^30^ We included experts with a professional background as well as with a patient background as recommended by COSMIN.^25^ Due to a large number of items, every expert rated one‐third of the item pool. The items were equally distributed according to the profession of the experts. For each item, experts were asked to rate two questions: ‘How relevant is this item for patients?’ (response scale 0 = not relevant, 1 = a little bit relevant, 2 = quite relevant, 3 = very relevant) and ‘How feasible is it for patients to assess this item?’ (response scale 0 = not feasible, 1 = feasible, but not easy, 2 = rather easy, 3 = easy). To check for comprehensiveness, we added a free text box for each dimension asking whether any important items were missing.

We established item deletion criteria in an exploratory approach after data collection using mean ratings and the content validity index (CVI). The CVI of each item is the percentage of experts who rated an item as ‘quite relevant’ or ‘very relevant’.^31^ In the literature, deletion of items with a CVI of less than 0.7 or 0.8 is recommended.^31^ We decided against such a high threshold as it would lead to the deletion of items that were not upmost but still quite relevant. Further, there is a discrepancy in recommendations that at least twice as many items as in the final questionnaire should be included in psychometric testing,^20^ and that each subscale needs to comprise at least four items.^32^ We decided to delete an item, if (1) the item was rated by at least half of the experts as not or hardly relevant (corresponding to a CVI of 0.5 or lower), (2) the item had mean feasibility to assess rating below 2 or (3) for subscales that still had more than four items, items with a mean relevance rating below 3 were deleted.

Finally, items were added after discussing the free text box comments in our team.

#### 2.5.3 Step 3.3: Checking comprehensibility

The resulting item pool was checked for comprehensibility in cognitive interviews with patients. To minimize the cognitive load of the questionnaire, we decided to use the same response scale for all items instead of changing it for different items. We translated a 4‐point scale describing frequencies into German (0 = never, 1 = sometimes, 2 = usually, 3 = always). This response scale is used for example in the Hospital Consumer Assessment of Healthcare Providers and Systems (HCAHPS).^33^

Participants in cognitive interviews were instructed to think of their last inpatient admission or outpatient appointment. The interviewer (S. Z.) asked participants to read the questions step by step and think aloud about how they would reply. Further, he asked them to explain certain words or phrases using their own words and about their preference regarding different versions of certain items. The interview guide was piloted with student research assistants and research interns and can be found in Appendix S4. Cognitive interviews were conducted by S. Z. without the presence of a third person. Some of the participants already knew the interviewer from the FGs, others met S. Z. for the first time. The interviews took place at the Department of Medical Psychology of the University Medical Center Hamburg‐Eppendorf (UKE) and were done in German.

Cognitive interviews were audio‐recorded, transcribed and anonymized and lasted between 45 and 60 min. The interviewer took field notes about the proposed changes directly after the interviews. Then, another researcher (E. C.) listened to the audio recordings, rated the comprehensibility of each item on a 3‐point scale (good, unclear, insufficient) and took notes on suggestions for item revisions made by the participants. The results and suggestions for item revisions were discussed by two researchers (S. Z., E. C.), consulting a third person if needed (I. S.). For each item, at least three cognitive interviews were conducted per round. If any of those interviews showed problems with the comprehensibility of an item or if any improvements were suggested, the corresponding item was adjusted and tested in another round. Only when all interviews in a round showed good comprehensibility of an item, we stopped testing it. Based on this procedure, we conducted two rounds of cognitive interviews as in the second round no items showed a further need for adjustments.

Participants who were willing to participate in a second interview round were allowed to do so as the recruitment process was hastened. In the second interview round, either completely new items were presented or items that were modified after the first round. The two interviews were at least 4 weeks apart.

## 3 RESULTS

An overview of the results is given in Figure 1.

### 3.1 Sample

We conducted six FGs with a total of 40 patients and 34 cognitive interviews with 21 patients (some participated in two interviews). We have no data on how many patients refused to participate. In cognitive interviews, there were no drop‐outs. In FGs, five patients did not show up without giving reasons.

Sample characteristics of the patients are shown in Table 1.

**Table 1:** Characteristics of the participants in the focus groups and cognitive interviews

Characteristics	Focus groups	Cognitive interviews
Sample size	40	21
Age (in years)	*M* = 54.2 (SD = 12.7), range: 30–79	*M* = 51.5 (SD = 15.0), range: 22–80
Sex		
Female	31 (77.5%)	16 (76.2%)
Male	9 (22.5%)	5 (23.8%)
First language		
German	38 (95.0%)	20 (95.2%)
Other	2 (5.0%)	1 (4.8%)
Occupational status^a^		
Employed	9 (22.5%)	7 (33.3%)
Unemployed	6 (15.0%)	5 (23.8%)
Retired	19 (47.5%)	7 (33.3%)
Student/trainee	2 (5.0%)	3 (14.3%)
Marital status		
Unmarried	25 (62.5%)	10 (47.6%)
Married/partnered	6 (15.0%)	3 (14.3%)
Divorced	9 (22.5%)	8 (38.1%)
Formal education		
Low^b^	6 (15%)	1 (4.8%)
Intermediate^c^	10 (25.0%)	8 (38.1%)
High^d^	15 (37.5%)	5 (23.8%)
Very high^e^	9 (22.5%)	7 (33.3%)
Chronic disease group^a^		
Cancer	5 (12.5%)	2 (9.5%)
Cardiovascular disease	10 (25.0%)	7 (33.3%)
Mental disorder	27 (67.5%)	13 (61.9%)
Musculoskeletal disorder	22 (55.0%)	12 (57.1%)

Abbreviations: *M*, mean; SD, standard deviation.

^a^ Multiple answers possible.

^b^ low = no formal degree or graduation after less than 10 years at school.

^c^ Intermediate = graduation after 10 or 11 years at school.

^d^ High = graduation after more than 11 years at school.

^e^ Very high = college or university degree.

For key informant interviews, 12 experts were invited of which 10 participated (two declined due to competing demands). There was no dropout. Mean age was 54.2 (standard deviation: 7.2, range: 44–65) and 80% were male. Five were physicians, two were working as head of a health facility/professional association and there was one support group coordinator, researcher and health insurance representative each.

Overall, 32 experts participated in the relevance rating. Each third of the items was rated by 8–13 experts from different professional backgrounds. The distribution of different professions over the items is reported in Table 2.

**Table 2:** Distribution of professions in the relevance rating (each one rating one‐third of the items)

	1st Third of items	2nd Third of items	3rd Third of items	Total
HCPs	3	3	4	11
Researchers	5	5	1	10
Patient representatives	3	4	3	10
Quality manager	0	1	0	1
Total	11	13	8	32

Abbreviation: HCPs, healthcare professionals.

### 3.2 Step 2: Item generation

We identified 48 internationally existing PREMs in the literature search (see Appendix S5 for references). Main settings were inpatient care (*n* = 7), primary care (*n* = 5) and other outpatient settings (*n* = 4). Most have been developed in Europe (*n* = 29) and North America (*n* = 17).

Based on the model, 16 codes corresponding to the 16 dimensions and 98 subcodes were derived (deductive approach). Based on the qualitative content analysis, we did not find any additional dimensions of PC but introduced 44 new subcodes (inductive approach). As we used convenience sampling, full theoretical saturation was not aimed for and was difficult to reach. Based on the last FG only two more codes were introduced. For a full overview of our coding tree and to know how many codes were derived from each source, please refer to Appendix S6. For text examples with the derived items and the full first item pool, please refer to Appendix S7. Overall, we developed 850 items, ranging from 15 items for the dimension ‘support of mental well‐being’ to 141 items for ‘PC characteristics of healthcare providers’.

### 3.3 Step 3: Item selection and testing of content validity

Deletion of repeating items as well as items that did not fit the context of use led to an item pool of 152 items ranging from three items for the dimension ‘support of mental well‐being’ to 15 items for the dimension ‘good planning of care’.

We deleted items as follows: (1) 7 items had a CVI of 0.5 or lower, (2) 5 items had a mean feasibility to assess rating below 2 and (3) 15 items had a mean relevance rating below 3.

Based on the free‐text comments in the relevance rating, we added four further items to the questionnaire.

This resulted in 129 items that were tested in cognitive interviews. Overall, item comprehensibility was high. Minor changes in 45 items were made after analysis of the first round. Those were changes of single words, addition of examples, or highlighting of single words. After the second round, no more adaptations were necessary.

Many participants considered the four response options derived from the HCAHPS too limiting and suggested adding more response categories (e.g., ‘often’ or ‘seldom’). We tested the suggested response categories in the second round of cognitive interviews and found that participants had differing opinions on which response options we should add and perceived the order of the new response categories differently. Therefore, we decided to use a 6‐point Likert scale rating agreement with statements instead (1 = completely disagree, 2 = strongly disagree, 3 = somewhat disagree, 4 = somewhat agree, 5 = strongly agree, 6 = completely agree). This response scale is used for example in the German SDM‐Q‐9 and SDM‐Q‐Doc,^34^, ^35^ a widely used^36^ questionnaire that was developed by team members and which was also transformed from a 4‐point‐scale to a 6‐point‐scale to reduce ceiling effects.^34^ While we did not test them with our questionnaire, cognitive interviews conducted at that time by a colleague (H. C., cp. acknowledgements) showed that patients were well able to use its response scale.

Based on the feedback of the participants, we deleted four items that were clearly dichotomous (e.g., ‘I received a discharge letter’) and did therefore not fit the response scale. As a result, the final item pool comprises 125 items. The inpatient version of the questionnaire has 121 items and the outpatient version has 120 items (with 116 items being identical for both versions).

## 4 DISCUSSION

### 4.1 Summary of results

We reported the process of the item development and selection of the EPAT questionnaire. It will be the first measure to assess all dimensions of the integrative model of PC^7^, ^8^ from the patients' perspective. Its current form has two versions, one for outpatient and one for inpatient healthcare while the items are the same for all four chronic disease groups considered here (cancer, cardiovascular disease, mental disorder, musculoskeletal disorder). All items in the final item pool showed good relevance and comprehensibility.

Our study offers a best‐practice example that other researchers may use to develop patient‐reported measures. In particular, we add to existing guidelines by describing a transparent process about how items were developed from qualitative data using a two‐step process. First, we used qualitative analysis to link the data to the construct definition and complemented the definition with the data. Second, we used the data to generate items for each dimension of the construct based on the context of use.

### 4.2 Strengths and limitations

A strength of this approach and the EPAT questionnaire is the involvement of patients in several steps: (1) in defining the construct to be measured, (2) during item generation using FGs and (3) during item selection in the relevance rating (as patient representatives) and in cognitive interviews. With these steps involving patients, we ensured that our item pool meets all three criteria given by the definition of content validity in the ‘COnsensus‐based Standards for the selection of health Measurement INstruments’ (COSMIN) criteria.^29^ This is highly relevant as content validity can be considered one of the most central psychometric properties of measures.^24^ To ensure high quality of patient‐reported measures, the input from patients is crucial as they are the primary experts regarding their own health and care.^25^ So far, many PREMs lack transparent reports of patient involvement. Two‐thirds of all PREMs identified by Bull and colleagues^13^ did not provide enough information to rate content validity even though they already used a simplified version of the COSMIN criteria. Other reviews on measures assessing subscales of PC resulted in mostly poor ratings of content validity, again, often due to missing information.^14^, ^15^, ^19^

One reason for such poor ratings might be that no guidelines exist for developing a PREM. This is also the reason why in this study we refer to references for patient‐reported outcome measures (PROMs). As both PREMs and PROMs are patient‐reported there are many similarities. Still, there is a big content‐related difference between assessing experience during a treatment (PREMs) or outcomes after a treatment (PROMs). Guidelines that take into account those content‐specific aspects for PREM development are much needed to foster the development of PREMs with high content validity.

A limitation of our study is that both patient samples are nonrepresentative regarding formal education. Further, the sample sizes in relevance rating and cognitive interviews are small. The COSMIN Study Design checklist recommends seven participants for qualitative studies while in our cognitive interviews, we had a minimum of three interviews per item. Having more participants was not feasible in this study due to the length of the item pool, resulting in a high number of cognitive interviews. Yet, we only stopped testing items if all three interviews showed good comprehensibility. All items that we adapted after the first round were tested in three more interviews and we stopped after that second round as all three interviews showed good comprehensibility.

Further limitations concern the literature search. As we lacked access to EMBASE, we searched only PubMed. COSMIN guidelines consider PubMed the minimum but still highly recommend including EMBASE.^37^ To compensate for that, we included reference tracking of a systematic review on PREMs, which used several databases.^12^ Another limitation is that references were screened by only one researcher. To reduce bias, this researcher included all measures she found during the screening. Whether these measures were relevant for our aim or not was decided during qualitative analysis where only relevant parts were coded and which was done by two researchers. For quantitative studies, the COSMIN checklist recommends a minimum of 50 participants while our items were rated by 8–13 experts. It has to be noted that this is above the minimum of 5–7 experts recommended by Boateng et al.^20^ Yet, a larger and more representative sample including not only patient representatives but also patients without a professional background in healthcare might have been beneficial and would have allowed for more rigorous decisions based on the relevance rating. In addition, while patients were included as a source for data collection, they were not actively involved in the research process. For future questionnaire developments including future research on the EPAT questionnaire, we suggest including patient researchers in the entire research process to enhance relevance.^38^ In particular, patients can be involved in setting priorities and defining measurement aims, designing guides for FGs and interviews, in the analysis of the qualitative data and in the item reduction process.

### 4.3 Implications for future research

The resulting item pool of the EPAT questionnaire is planned to be psychometrically tested with *n* = 2000 patients from the four chronic disease groups in inpatient and outpatient settings.^23^ We will examine item characteristics like floor and ceiling effects, the number of missing values and corrected item‐total correlations as well as the factor structure to further reduce the item pool. We aim to select one item for each of the 16 dimensions to obtain a short version of the questionnaire that can be used easily in routine care. Further items will be available to assess each dimension in more detail. This will allow users (e.g., researchers, HCPs, healthcare managers, etc.) to select parts of the questionnaire depending on their aim of measurement, their focus of interest and their available resources.

Eventually, the EPAT can be used to examine the effectiveness of interventions aiming to promote PC or certain dimensions of PC in Germany.

Furthermore, it can be used to explore relationships between the level of PC (e.g., of a healthcare centre or a single ward or HCP) and other outcomes like patient‐reported outcomes (e.g., health‐related quality of life).

The items of the EPAT were derived for four different chronic disease groups regardless of the different treatments, symptoms and so forth, and thus are roughly disease generic. The items should be tested with patients with further chronic diseases as well as acute diseases to investigate whether the EPAT can be used for them as well. So far, the EPAT will be available only in German. After the questionnaire is finalized we recommend it to be translated, culturally adapted and tested with patient groups in other countries. To our knowledge, there is no measure in any language that assesses PC directly from the patients' view in such detail while at the same time allowing users to adapt it flexibly to their own needs. As the model of PC that was used for development is based on international literature, we expect many items to be independent of the German context. Translating the EPAT questionnaire into other languages would allow for the comparison of PC in different countries and healthcare systems.

Moreover, the EPAT could be translated for migrant groups living and experiencing the healthcare system in Germany or adapted for other patient groups (e.g., patients with a disability). Hereby, special PCs needs or barriers for more vulnerable groups could be detected and addressed.

Finally, the EPAT could also be adapted to measure the perspective of HCPs on PC processes within their organization, at their workplace or in their patient contacts or on their perceived own competencies in PC. Therefore, the patients and the HCP perspective could be compared and PC needs on both sides could be detected. Possible results could be used to develop PC training and interventions to foster PC (e.g., to strengthen the perceived self‐efficacy or competencies of HCP or to change work processes within organizations).

## 5 CONCLUSIONS

We developed a preliminary version of the EPAT questionnaire, which will undergo psychometric testing. It can be used to assess PC from the patients' perspective in inpatient and outpatient healthcare. The thorough development process that included patients in several steps ensures good content validity of the EPAT questionnaire and can be used as a best‐practice example to develop patient‐reported measures from qualitative data.

## AUTHOR CONTRIBUTIONS

Isabelle Scholl and Jördis Zill were the responsible principal investigators of the study. Isabelle Scholl, Levente Kriston and Martin Härter were involved in the planning and preparation of the study. Eva Christalle and Stefan Zeh recruited participants and collected data. Eva Christalle, Stefan Zeh and Jördis Zill analysed data. Eva Christalle, Stefan Zeh, Jördis Zill, Isabelle Scholl, Pola Hahlweg and Levente Kriston interpreted the results. Eva Christalle wrote the first draft of the manuscript. All authors critically revised the manuscript for important intellectual content. All authors gave final approval of the version to be published and agreed to be accountable for the work.

## CONFLICTS OF INTEREST

The authors declare no conflicts of interest.

## ETHICS STATEMENT

The study was carried out according to the latest version of the Helsinki Declaration of the World Medical Association. Principles of good scientific practice were respected. The study had been approved by the Ethics Committee of the Medical Association Hamburg (study ID: PV5724). Study participation was voluntary and no foreseeable risks for participants resulted from the participation in this study. Participants were fully informed about the aims of the study, data collection and the use of collected data. Written informed consent was obtained before participation. Preserving principles of data sensitivity, data protection and confidentiality requirements were met.

## Supporting information

Supporting information.

Supporting information.

Supporting information.

Supporting information.

Supporting information.

Supporting information.

Supporting information.

## Data Availability

Deidentified data that support the findings of this study are available on reasonable request. Investigators who propose to use the data have to provide a methodologically sound proposal directed to the corresponding author. Signing a data use/sharing agreement will be necessary, and data security regulations both in Germany and in the country of the investigator who proposes to use the data must be complied with. Preparing the data set for use by other investigators requires substantial work and is thus linked to available or provided resources. The data sets used in this study are in the German language.

## References

[1] Dwamena F , Holmes‐Rovner M , Gaulden CM , et al. Interventions for providers to promote a patient‐centred approach in clinical consultations. Cochrane Database Syst Rev . 2012;(12). 10.1002/14651858.CD003267.pub2 PMC9947219 23235595

[2] McMillan SS , Kendall E , Sav A , et al. Patient‐centered approaches to health care: a systematic review of randomized controlled trials. Med Care Res Rev. 2013;70(6):567‐596. 23894060 10.1177/1077558713496318

[3] Rathert C , Wyrwich MD , Boren SA . Patient‐centered care and outcomes: a systematic review of the literature. Med Care Res Rev. 2013;70(4):351‐379. 23169897 10.1177/1077558712465774

[4] Berwick DM . A user's manual for the IOM's ‘Quality Chasm’ report. Health Aff. 2002;21(3):80‐90. 10.1377/hlthaff.21.3.80 12026006

[5] World Health Organization (WHO) . Innovative Care For Chronic Conditions: Building Blocks For Action.2002. Accessed May 17, 2021. https://apps.who.int/iris/bitstream/handle/10665/42500/WHO_NMC_CCH_02.01.pdf

[6] Deutscher Bundestag . Gesetz zur Verbesserung der Rechte von Patientinnen und Patienten. Bundesgesetzblatt. 2013;​(Teil I):277‐282.

[7] Scholl I , Zill JM , Härter M , Dirmaier J . An integrative model of patient‐centeredness–a systematic review and concept analysis. PLoS One. 2014;9(9):e107828. 25229640 10.1371/journal.pone.0107828 PMC4168256

[8] Zeh S , Christalle E , Hahlweg P , Härter M , Scholl I . Assessing the relevance and implementation of patient‐centredness from the patients' perspective in Germany: results of a Delphi study. BMJ Open. 2019;9(12):e031741. 10.1136/bmjopen-2019-031741 PMC7008421 31874875

[9] De Silva D . Helping measure person‐centred care: a review of evidence about commonly used approaches and tools used to help measure person‐centred care. 2014. Accessed May 17, 2021. https://www.health.org.uk/publications/helping-measure-person-centred-care

[10] Berwick DM , Nolan TW , Whittington J . The triple aim: care, health, and cost. Health Aff. 2008;27(3):759‐769. 10.1377/hlthaff.27.3.759 18474969

[11] Bodenheimer T , Sinsky C . From triple to quadruple aim: care of the patient requires care of the provider. Ann Fam Med. 2014;12(6):573‐576. 25384822 10.1370/afm.1713 PMC4226781

[12] Beattie M , Murphy DJ , Atherton I , Lauder W . Instruments to measure patient experience of healthcare quality in hospitals: a systematic review. Syst Rev. 2015;4(1):1‐21. 26202326 10.1186/s13643-015-0089-0 PMC4511995

[13] Bull C , Byrnes J , Hettiarachchi R , Downes M . A systematic review of the validity and reliability of patient‐reported experience measures. Health Serv Res. 2019;54(5):1023‐1035. 31218671 10.1111/1475-6773.13187 PMC6736915

[14] Barr PJ , Scholl I , Bravo P , Faber MJ , Elwyn G , McAllister M . Assessment of patient empowerment‐a systematic review of measures. PLoS One. 2015;10(5):e0126553. 25970618 10.1371/journal.pone.0126553 PMC4430483

[15] Christalle E , Zill JM , Frerichs W , et al. Assessment of patient information needs: a systematic review of measures. PLoS One. 2019;14(1):e0209165. 30703103 10.1371/journal.pone.0209165 PMC6354974

[16] Gärtner FR , Bomhof‐Roordink H , Smith IP , Scholl I , Stiggelbout AM , Pieterse AH . The quality of instruments to assess the process of shared decision making: a systematic review. PLoS One. 2018;13(2):e0191747. 29447193 10.1371/journal.pone.0191747 PMC5813932

[17] Müller E , Zill JM , Dirmaier J , Härter M , Scholl I . Assessment of trust in physician: a systematic review of measures. PLoS One. 2014;9(9):e106844. 25208074 10.1371/journal.pone.0106844 PMC4160203

[18] Sepucha KR , Scholl I . Measuring shared decision making: a review of constructs, measures, and opportunities for cardiovascular care. Circ Cardiovasc Qual Outcomes. 2014;7(4):620‐626. 24867916 10.1161/CIRCOUTCOMES.113.000350

[19] Zill JM , Christalle E , Müller E , Härter M , Dirmaier J , Scholl I . Measurement of physician‐patient communication—a systematic review. PLoS One. 2014;9(12):e112637. 25532118 10.1371/journal.pone.0112637 PMC4273948

[20] Boateng GO , Neilands TB , Frongillo EA , Melgar‐Quiñonez HR , Young SL . Best practices for developing and validating scales for health, social, and behavioral research: a primer. Front Public Health. 2018;6:149. 29942800 10.3389/fpubh.2018.00149 PMC6004510

[21] Mokkink LB , Prinsen CA , Patrick DL , et al. COSMIN Study Design checklist for Patient‐reported outcome measurement instruments.2019. Accessed May 17, 2021. https://www.cosmin.nl/wp-content/uploads/COSMIN-study-designing-checklist_final.pdf

[22] Streiner DL , Norman GR , Cairney J . Health Measurement Scales: a Practical Guide to their Development and Use. Oxford University Press; 2015.

[23] Christalle E , Zeh S , Hahlweg P , Kriston L , Härter M , Scholl I . Assessment of patient centredness through patient‐reported experience measures (ASPIRED): protocol of a mixed‐methods study. BMJ Open. 2018;8(10):e025896 10.1136/bmjopen-2018-025896 PMC6196960 30344183

[24] Terwee CB , Prinsen CA , Chiarotto A , et al. COSMIN methodology for assessing the content validity of PROMs User manual version 1.0. 2021. Accessed May 19, 2021. https://cosmin.nl/wp-content/uploads/COSMIN-methodology-for-content-validity-user-manual-v1.pdf

[25] Terwee CB , Prinsen C , Chiarotto A , et al. COSMIN methodology for evaluating the content validity of patient‐reported outcome measures: a Delphi study. Qual Life Res. 2018;27(5):1159‐1170. 29550964 10.1007/s11136-018-1829-0 PMC5891557

[26] VERBI Software . MAXQDA. VERBI Software, 2017. 2018.

[27] Mayring P . Qualitative content analysis. Forum: Qualitative Social Research. 2000;1(2):Art. 20.

[28] Mayring P . *Qualitative content analysis: theoretical foundation, basic procedures and software solution. * 2014.

[29] Prinsen C , Mokkink LB , Bouter LM , et al. COSMIN guideline for systematic reviews of patient‐reported outcome measures. Qual Life Res. 2018;27(5):1147‐1157. 29435801 10.1007/s11136-018-1798-3 PMC5891568

[30] LimeSurvey GmbH, Hamburg, Germany . Accessed April 11, 2022. http://www.limesurvey.org

[31] Polit DF , Beck CT . The content validity index: are you sure you know what's being reported? Critique and recommendations. Res Nurs Health. 2006;29(5):489‐497. 16977646 10.1002/nur.20147

[32] Diamantopoulos A , Sarstedt M , Fuchs C , Wilczynski P , Kaiser S . Guidelines for choosing between multi‐item and single‐item scales for construct measurement: a predictive validity perspective. J Acad Market Sci. 2012;40(3):434‐449.

[33] Giordano LA , Elliott MN , Goldstein E , Lehrman WG , Spencer PA . Development, implementation, and public reporting of the HCAHPS survey. Med Care Res Rev. 2010;67(1):27‐37. 19638641 10.1177/1077558709341065

[34] Kriston L , Scholl I , Hölzel L , Simon D , Loh A , Härter M . The 9‐item Shared Decision Making Questionnaire (SDM‐Q‐9). Development and psychometric properties in a primary care sample. Patient Educ Couns. 2010;80(1):94‐99. 19879711 10.1016/j.pec.2009.09.034

[35] Scholl I , Kriston L , Dirmaier J , Buchholz A , Härter M . Development and psychometric properties of the Shared Decision Making Questionnaire–physician version (SDM‐Q‐Doc). Patient Educ Couns. 2012;88(2):284‐290. 22480628 10.1016/j.pec.2012.03.005

[36] Doherr H , Christalle E , Kriston L , Härter M , Scholl I . Use of the 9‐item Shared Decision Making Questionnaire (SDM‐Q‐9 and SDM‐Q‐Doc) in intervention studies—a systematic review. PLoS One. 2017;12(3):e0173904. 28358864 10.1371/journal.pone.0173904 PMC5373562

[37] Prinsen CA , Vohra S , Rose MR , et al. Guideline for Selecting Outcome Measurement Instruments for Outcomes Included in a Core Outcome Set. COMET COSMIN; 2016. 10.1186/s13063-016-1555-2 PMC5020549 27618914

[38] Greenhalgh T , Hinton L , Finlay T , et al. Frameworks for supporting patient and public involvement in research: systematic review and co‐design pilot. Health Expect. 2019;22(4):785‐801. 31012259 10.1111/hex.12888 PMC6737756

