# Supplementary material for: Development and content validity of the Experienced Patient‐Centeredness Questionnaire (EPAT)—A best practice example for generating patient‐reported measures from qualitative data
Source: Health Expect. 2022 Apr 21;25(4):1529–38. doi: 10.1111/hex.13494 (PMC9327838; doi:10.1111/hex.13494)
Supplement: Supplementary file 1 — Supporting information. [file HEX-25--s004.docx]

**Appendix 1: COREQ Checklist** [1]

| **Domain 1: Research team and reflexivity** | | |
| --- | --- | --- |
| **Personal Characteristics** | | |
| **1. Interviewer/facilitator** | Which author/s conducted the interview or focus group? | Page 8 and 12 |
| **2. Credentials** | What were the researcher’s credentials? E.g. PhD, MD | Page 8 |
| **3. Occupation** | What was their occupation at the time of the study? | Page 8 |
| **4. Gender** | Was the researcher male or female? | Page 8 |
| **5. Experience and training** | What experience or training did the researcher have? | Page 8 |
| **Relationship with participants** | | |
| **6. Relationship established** | Was a relationship established prior to study commencement? | Page 8 |
| **7. Participant knowledge of the interviewer** | What did the participants know about the researcher? e.g. personal goals, reasons for doing the research | Page 8 and 12 |
| **8. Interviewer characteristics** | What characteristics were reported about the interviewer/facilitator? e.g. Bias, assumptions, reasons and interests in the research topic | Page 8 |
| **Domain 2: study design** | | |
| **Theoretical framework** | | |
| **9. Methodological orientation and Theory** | What methodological orientation was stated to underpin the study? e.g. grounded theory, discourse analysis, ethnography, phenomenology, content analysis | Page 9 |
| **Participant selection** | | |
| **10. Sampling** | How were participants selected? e.g. purposive, convenience, consecutive, snowball | Page 5 |
| **11. Method of approach** | How were participants approached? e.g. face-to-face, telephone, mail, email | Page 5 |
| **12. Sample size** | How many participants were in the study? | Table 1 and 2 |
| **13. Non-participation** | How many people refused to participate or dropped out? Reasons? | Page 14 |
| **Setting** | | |
| **14. Setting of data collection** | Where was the data collected? e.g. home, clinic, workplace | Page 8 and 12 |
| **15. Presence of non-participants** | Was anyone else present besides the participants and researchers? | Page 8 and 12 |
| **16. Description of sample** | What are the important characteristics of the sample? e.g. demographic data, date | Table 1 and 2 |
| **Data** **collection** | | |
| **17. Interview guide** | Were questions, prompts, guides provided by the authors? Was it pilot tested? | Appendix 2 to 4 and page 8 and 12 |
| **18. Repeat interviews** | Were repeat interviews carried out? If yes, how many? | Page 9 |
| **19. Audio/visual recording** | Did the research use audio or visual recording to collect the data? | Page 9 and 13 |
| **20. Field notes** | Were field notes made during and/or after the interview or focus group? | Page 9 |
| **21. Duration** | What was the duration of the interviews or focus group? | Page 9 |
| **22. Data saturation** | Was data saturation discussed? | Page 15 |
| **23. Transcripts returned** | Were transcripts returned to participants for comment and/or correction? | Page 9 |
| **Domain 3: analysis and findings** | | |
| **Data analysis** | | |
| **24. Number of data coders** | How many data coders coded the data? | Page 9 |
| **25. Description of the coding tree** | Did authors provide a description of the coding tree? | Appendix 6 |
| **26. Derivation of themes** | Were themes identified in advance or derived from the data? | Page 9 |
| **27. Software** | What software, if applicable, was used to manage the data? | Page 9 and 10 |
| **28. Participant checking** | Did participants provide feedback on the findings? | Page 9 |
| **Reporting** | | |
| **29. Quotations presented** | Were participant quotations presented to illustrate the themes / findings? Was each quotation identified? e.g. participant number | Appendix 7 |
| **30. Data and findings consistent** | Was there consistency between the data presented and the findings? | Appendix 6 and 7 |
| **31. Clarity of major themes** | Were major themes clearly presented in the findings? | Page 10 |
| **32. Clarity of minor themes** | Is there a description of diverse cases or discussion of minor themes? | Page 10 |

1. Tong A, Sainsbury P, Craig J. Consolidated criteria for reporting qualitative research (COREQ): a 32-item checklist for interviews and focus groups. *International journal for quality in health care* 2007;19(6):349-57.
