# Supplementary material for: Development and content validity of the Experienced Patient‐Centeredness Questionnaire (EPAT)—A best practice example for generating patient‐reported measures from qualitative data
Source: Health Expect. 2022 Apr 21;25(4):1529–38. doi: 10.1111/hex.13494 (PMC9327838; doi:10.1111/hex.13494)
Supplement: Supplementary file 2 — Supporting information. [file HEX-25--s007.docx]

**Appendix 2: Guide for focus groups**

***[original version in German]***

**General concept:**

- Objective: To identify which aspects and, where appropriate, specific actions are particularly important for the participants in the provision of care.
- Protocol, technology & assistance in the organizational process by interns / student research assistants
- Characteristics of the moderator
  - Dynamic, non-authoritarian, honest and calm, good time manager, flexible but prepared, reflective and responsive.
- Behavior of the moderator
  - Do not interrupt, be patient, enable rather than dominate, ask rather than tell, listen carefully, do not evaluate or judge, do not fight the group, disruptions have priority, do not discuss the method, distinguish between perceptions, assumptions and evaluations
  - Friendly-distant, authoritative
  - Screen dissenting opinions when group opinion is formed
  - Return questions to the group or refer to a later point in the group discussion
  - Own opinions and knowledge of the moderator must not play a role
  - Avoid leading questions
  - Shy participants & experts - include or slow down
- Language, phrases of the moderator
  - Reflect language of the participants adapted in age, gender and position
  - Paraphrasing in words/phrases used by participants, no re-interpretation.
- Aim of the moderation
  - Generation of detailed representations (helpful resources: direct questions about narratives, descriptions, the experience, series of questions)
  - Collect data as objectively as possible, i.e., interview without interpretation or preconceived ideas

**Focus group procedure:**

Prepared in advance, one stack per participant with: **Demographic questionnaire, expense reimbursement form, name cards, consent form**, **envelope.**

**Prepare questions on flipchart**

**There are pens, moderation cards and adhesive dots at the place**

**Introduction (10 minutes)**

Say hello and thank you.

**Introduction**

- Presentation of the Institute for Medical Psychology
- Introduction of the moderators and the keeper of the minutes
- Presentation of research group and project
- Aim of the focus group: Report experiences with the health care system and tell what is particularly important about it.
- We want to develop questions for a questionnaire based on the results of the discussion. The questionnaire asks about the quality of health care from the patients' perspective. It is important that the questions are relevant for patients. In this way, the questionnaire can help to improve the health care system.
- Duration: 90-120 minutes
- In between 10 minutes break

**Structure of the group discussion**

- Tasks of the moderator:
  - We structure the discussion and ask questions.
  - At the beginning we collect a few points on the pin board at the front and try to summarize topics.
  - Otherwise, we tend to stay in the background.
  - We may intervene if you stray from the topic.
- Expectations for participants:
  - Participants are the experts 🡪 „You discuss”
  - express personal opinions and experiences
  - "Everyone should have their say on every question"
- No right or wrong 🡪 “We are interested in all opinions and experiences - both critical and positive comments." "You don't have to agree."

**Address again:**

- Audio recording of the discussion (transcription + anonymization + deletion of recordings after data analysis) 🡪OK with everyone?
- Completion of a short questionnaire with information about the person and illness and declaration of consent and compensation for expenses

**Round of introductions (5-10 minutes)**

Name; if desired: disease, which treatments (1 sentence, keep short)

**Discussion (40 minutes)**

- As I said earlier, today is supposed to be about your own experiences in the health care system.
- We focus on what is particularly important for you. It is about what was or is good as well as what should have been done differently.

First of all, let's give you some time right now to collect a few ideas on these two questions on your own. For this purpose, you have various cards and pens in front of you. Please write down key points on the following questions. Afterwards we will collect your cards here in front and discuss them together. We will try to sort them by topic.

*[Flipchart + cards and pens are ready]*

**Flipchart:**

- *"What did you find or do you find good about your health care?"*
- *"What should have been done differently?"*

Think about your own experiences in your health care when replying to the questions. You can refer to all areas of your health care, e.g., a conversation with a doctor, the overall situation in a hospital or a practice, or treatment by health professionals who are not doctors, such as nurses, psychotherapists, physiotherapists, occupational therapists, etc.

Think about this for a moment:

1. What have you found or do you find particularly good in your health care?
2. What should have been different during your health care?

Please write down three important positive and three important negative experiences that you have had. You may bring in all the memories you can remember. There is no right or wrong here.

*[give them 7 minutes]*

*[from here on approx. 30 minutes time for collecting and sorting]*

We want to start collecting the topics now. Did you have enough time? *[give more time if necessary]*

Good, then we collect the topics here in the front. Please say a few sentences about your card, what exactly you mean by it.

Who wants to start with a card?

*[Moderator collects topics, tries to group them. The moderator invites the whole group to add topics from their cards instead of working through the cards person by person.]*

Did anyone else write down something on this topic?

Has anyone written down a similar point?

Anyone else wants to add a card?

*[when discussion dies down] Did* anyone else write down a point we haven't mentioned yet?

**Break (10 minutes)**

**Discussion (20 minutes)**

*[Discussion continues as above]*

**Summary (5 minutes)**

*[First summarize the reports and describe the relevance, then mention the most important points and subsume concrete behaviors.]*

Is there something we missed?

Is there something we should have talked about?

**Conclusion (5 minutes)**

Collect expense reimbursement form + questionnaire + consent form

Acknowledgement and farewell
