# Supplementary material for: Development and content validity of the Experienced Patient‐Centeredness Questionnaire (EPAT)—A best practice example for generating patient‐reported measures from qualitative data
Source: Health Expect. 2022 Apr 21;25(4):1529–38. doi: 10.1111/hex.13494 (PMC9327838; doi:10.1111/hex.13494)
Supplement: Supplementary file 3 — Supporting information. [file HEX-25--s001.docx]

**Appendix 3: Guide for key informant interviews**

***[original version in German]***

**Introduction**

*Say hello and thank you.*

Thank you for agreeing to participate in this interview.

As you already know, it is about patient-centeredness from the patient's perspective. To this end, we are developing a questionnaire that can be used to assess patients' experiences, thereby enabling an improvement in the quality of care in Germany. Therefore, we would like to try to develop questions for our questionnaire together with you in an interview.

The interview will take about 30-45 minutes. Everything you say here will be analyzed anonymously. This means that no data will be collected that allows to identify you. For our analysis, we will record the interview using this small audio device. Do you agree to this?

At the end of the interview I will ask you to fill out a short questionnaire with general information. This data won’t allow any conclusions about your person either and will be evaluated independently of the interview.

If you wish to do so, you can stop the interview at any time without having any disadvantages.

During the interview we will work through individual dimensions of patient-centeredness together. For this purpose, I will ask you questions about what has to be done specifically so that patient-centeredness can be experienced by patients. This can include, for example, specific actions or behaviors of the practitioners. In addition, I will ask follow-up questions in order to understand your thoughts as precisely as possible.

You are welcome to say out loud everything that goes through your mind and to "think aloud", so to speak. There is no right or wrong here, only your own thoughts.

Do you have any questions?

***[Example for one dimension shown to the participant]***

**Patient-centered characteristics of the practitioners**

The practitioners are:

- empathetic
- honest
- appreciative
- trustworthy

The practitioners are aware of their own behavior and emotions. The practitioners have good professional competence.

***[Questions asked for this dimension]***

What does the treatment have to look like for this characteristic to be fulfilled?

What behavior would the practitioners have to show in order for this dimension to be implemented?

What patient-centered characteristics would practitioners further need to demonstrate?

What are the characteristics of a patient-centered treatment?
