# Supplementary material for: Development and content validity of the Experienced Patient‐Centeredness Questionnaire (EPAT)—A best practice example for generating patient‐reported measures from qualitative data
Source: Health Expect. 2022 Apr 21;25(4):1529–38. doi: 10.1111/hex.13494 (PMC9327838; doi:10.1111/hex.13494)
Supplement: Supplementary file 4 — Supporting information. [file HEX-25--s005.docx]

**Appendix 4: Guide for cognitive interviews**

***[original version in German]***

**Introduction**

Thank you for agreeing to participate in this interview. As you have already read in the information sheet on the study, our team has developed a questionnaire on the subject of patient-centeredness. The aim of this interview is to find out whether the statements are well understandable.

The interview will take about 45-60 minutes. Everything you say here will be analyzed anonymously. This means that no data will be collected that would allow to identify you. For our evaluation we will record the interview using this small audio device. We transcribe the audio recording, deleting all names of persons, places and the like, so that the transcription is anonymous. Do you agree with this?

At the end of the interview I will ask you to fill out a short questionnaire with general information. This data will also not allow any conclusions to be drawn about your person and will be evaluated independently of the interview.

Let us discuss your task in the interview: As I said, our topic is whether the statements are well understandable.

I would like to ask you to go through the statements in this questionnaire with me bit by bit and to think aloud. Tell me everything that strikes you, how you understand the question and anything else that occurs to you.

I will help you think aloud by asking a few questions such as "How do you understand the question?", "How would you reply to the question if you were thinking about your last doctor's appointment?", or "What reasons can you think of why you would have answered the question this way?".

At some points I will also ask you to rephrase individual words or I will ask you questions about them. I will often ask you to describe in your own words what you have just read. This is very unusual at first, but it is important for me to be able to understand how the text is understood by you.

You are welcome to speak out loud everything that goes through your mind. There is no right or wrong here, only your own thoughts.

It is important to keep in mind that this interview is not about whether you like or dislike the questions, but only about whether you understand the content.

If you wish to do so, you can stop the interview at any time without having any disadvantages.

Do you have any questions?

Now, this is the questionnaire. *[hand out questionnaire]* We will now go through the questionnaire together statement by statement.

In this questionnaire, you will often read the term [practitioner]. This is to be understood as a placeholder. Depending on where you are being treated, the term can be replaced with "the doctor", "the treatment team" or "the nursing staff", for example. When answering the questions, please think about your last appointment with your practitioners or your last hospital stay. So if it was with a GP, for example, think of your appointment with that GP at that point.

Do you have a situation in mind right now that you could evaluate? - Then refer to *[insert what is reported by the patient].*

You will also see the different answer options. These range from "never" to "always" to assess how much these statements apply.

Another answer option is "does not concern me". Tick this answer if the question does not apply to your treatment.

Do you have any questions before we start?

*[turn on audio device]*

***[Prompts used for all items]***

How do you understand this question?

What does the word XXX mean to you?

Please explain in your own words what is meant by XXX from your point of view.

How would you reply to the following question?

What would you tick here in the question?

What are the reasons that you would have ticked here?

**First question:** "Doesn't concern me"

- Here, point out the answer "does not concern me" and ask for explanation of what is meant by this.
- Alternative to "does not concern me": "not applicable" - what is meant by this? Which alternative is better?

***[Items for one example dimension with additional questions to be asked]***

**Patient-centered characteristics of the practitioners**

The treating persons behaved in a respectful and appreciative way.

*Test “dignifiedly” E.g. How would you understand the word “dignifiedly”? What does “dignifiedly” mean to you?*

I was taken seriously.

The practitioners were empathetic (for example, they responded to my feelings, showed understanding, or empathized with my situation).

*Are the examples here good for understanding the word "empathetic"?*

Those treating me talked about me in my presence as if I hadn't been there. *[Only hospital stay]*

The practitioners were committed to finding a solution to my health concerns.

*What does the word “committed” mean to you?*

*Alternative versions:*

- *The practitioners were motivated to find a solution to my health problems.*
- *The doctors made an effort to find a solution for my health problems.*

*Test both versions and ask which one is easier to understand.*

Difficult topics were discussed directly and openly by the practitioners (for example, long-term consequences of the disease, life expectancy or sexuality).

I felt I was given honest and sincere advice.

*What is the difference between honest and sincere? Same content?*

*If someone wants to enrich themselves financially instead of really helping you - would you understand this behavior as well?*
