# Supplementary material for: Development and content validity of the Experienced Patient‐Centeredness Questionnaire (EPAT)—A best practice example for generating patient‐reported measures from qualitative data
Source: Health Expect. 2022 Apr 21;25(4):1529–38. doi: 10.1111/hex.13494 (PMC9327838; doi:10.1111/hex.13494)
Supplement: Supplementary file 5 — Supporting information. [file HEX-25--s003.docx]

**Appendix 5: Measures included in literature search**

1. Arnetz, J. E., Höglund, A. T., Arnetz, B. B., & Winblad, U. (2008). Development and evaluation of a questionnaire for measuring patient views of involvement in myocardial infarction care. European Journal of Cardiovascular Nursing, 7(3), 229-238.
2. Barr, P. J., Forcino, R. C., Thompson, R., Ozanne, E. M., Arend, R., Castaldo, M. G., ... & Elwyn, G. (2017). Evaluating CollaboRATE in a clinical setting: analysis of mode effects on scores, response rates and costs of data collection. BMJ open, 7(3).
3. Beattie, M., Shepherd, A., Lauder, W., Atherton, I., Cowie, J., & Murphy, D. J. (2016). Development and preliminary psychometric properties of the Care Experience Feedback Improvement Tool (CEFIT). BMJ open, 6(6), e010101.
4. Bjertnaes, O., Iversen, H. H., Holmboe, O., Danielsen, K., & Garratt, A. (2016). The Universal Patient Centeredness Questionnaire: reliability and validity of a one-page questionnaire following surveys in three patient populations. Patient related outcome measures, 7, 55.
5. Bobrovitz, N., Santana, M. J., Ball, C. G., Kortbeek, J., & Stelfox, H. T. (2012). The development and testing of a survey to measure patient and family experiences with injury care. Journal of Trauma and Acute Care Surgery, 73(5), 1332-1339. *[two measures]*
6. Bobrovitz, N., Santana, M., Kline, T., Kortbeek, J., & Stelfox, H. T. (2013). Prospective cohort study protocol to evaluate the validity and reliability of the Quality of Trauma Care Patient-Reported Experience Measure (QTAC-PREM). BMC health services research, 13(1), 1-8.
7. Bos, N., Sizmur, S., Graham, C., & van Stel, H. F. (2013). The accident and emergency department questionnaire: a measure for patients’ experiences in the accident and emergency department. BMJ quality & safety, 22(2), 139-146.
8. Bos, N., Sturms, L. M., Schrijvers, A. J., & van Stel, H. F. (2012). The consumer quality index (CQ-index) in an accident and emergency department: development and first evaluation. BMC health services research, 12(1), 1-10.
9. Bosworth, A., Cox, M., O’Brien, A., Jones, P., Sargeant, I., Elliott, A., & Bukhari, M. (2015). Development and validation of a Patient Reported Experience Measure (PREM) for patients with rheumatoid arthritis (RA) and other rheumatic conditions. Current rheumatology reviews, 11(1), 1-7.
10. Brannan, Ana Maria, Susan Elizabeth Sonnichsen, and Craig Anne Heflinger. "Measuring satisfaction with children's mental health services: Validity and reliability of the satisfaction scales." Evaluation and program planning 19.2 (1996): 131-141.
11. Brown, A., Ford, T., Deighton, J., & Wolpert, M. (2014). Satisfaction in child and adolescent mental health services: Translating users’ feedback into measurement. Administration and Policy in Mental Health and Mental Health Services Research, 41(4), 434-446.
12. Casu, G., Gremigni, P., & Sommaruga, M. (2019). The Patient-Professional Interaction Questionnaire (PPIQ) to assess patient centered care from the patient’s perspective. Patient education and counseling, 102(1), 126-133.
13. Cinocca, S., Rucci, P., Randazzo, C., Teale, E., Pianori, D., Ciotti, E., & Fantini, M. P. (2017). Validation of the italian version of the Patient reported experience Measures for intermediate care services. Patient preference and adherence, 11, 1671.
14. Cleary, P. D., Edgman-Levitan, S., Roberts, M., Moloney, T. W., McMullen, W., Walker, J. D., & Delbanco, T. L. (1991). Patients evaluate their hospital care: a national survey. Health Affairs, 10(4), 254-267.
15. Davies, E., Shaller, D., Edgman‐Levitan, S., Safran, D. G., Oftedahl, G., Sakowski, J., & Cleary, P. D. (2008). Evaluating the use of a modified CAHPS® survey to support improvements in patient‐centred care: lessons from a quality improvement collaborative. Health Expectations, 11(2), 160-176.
16. Day, C., Michelson, D., & Hassan, I. (2011). Child and adolescent service experience (ChASE): Measuring service quality and therapeutic process. British Journal of Clinical Psychology, 50(4), 452-464.
17. De Witte, L., Schoot, T., & Proot, I. (2006). Development of the client‐centred care questionnaire. Journal of advanced nursing, 56(1), 62-68.
18. Dyer, N., Sorra, J. S., Smith, S. A., Cleary, P., & Hays, R. (2012). Psychometric properties of the Consumer Assessment of Healthcare Providers and Systems (CAHPS®) clinician and group adult visit survey. Medical care, 50(Suppl), S28.
19. Farin, E., Gramm, L., & Kosiol, D. (2011). Development of a questionnaire to assess communication preferences of patients with chronic illness. Patient Education and Counseling, 82(1), 81-88.
20. Frank, C., Asp, M., Fridlund, B., & Baigi, A. (2011). Questionnaire for patient participation in emergency departments: development and psychometric testing. Journal of advanced nursing, 67(3), 643-651.
21. Garratt, A. M., Bjærtnes, Ø. A., Krogstad, U., & Gulbrandsen, P. (2005). The OutPatient Experiences Questionnaire (OPEQ): data quality, reliability, and validity in patients attending 52 Norwegian hospitals. BMJ Quality & Safety, 14(6), 433-437.
22. Giordano, L. A., Elliott, M. N., Goldstein, E., Lehrman, W. G., & Spencer, P. A. (2010). Development, implementation, and public reporting of the HCAHPS survey. Medical Care Research and Review, 67(1), 27-37.
23. Glasgow, R. E., Wagner, E. H., Schaefer, J., Mahoney, L. D., Reid, R. J., & Greene, S. M. (2005). Development and validation of the patient assessment of chronic illness care (PACIC). Medical care, 436-444.
24. Gore, C., Griffin, R., Rothenberg, T., Tallett, A., Hopwood, B., Sizmur, S., ... & Warner, J. O. (2016). New patient-reported experience measure for children with allergic disease: development, validation and results from integrated care. Archives of disease in childhood, 101(10), 935-943.
25. Gutteling, J. J., De Man, R. A., Busschbach, J. J., & Darlington, A. S. E. (2008). Quality of health care and patient satisfaction in liver disease: the development and preliminary results of the QUOTE-Liver questionnaire. BMC gastroenterology, 8(1), 1-7.
26. Haggerty, J. L., Roberge, D., Freeman, G. K., Beaulieu, C., & Bréton, M. (2012). Validation of a generic measure of continuity of care: when patients encounter several clinicians. The Annals of Family Medicine, 10(5), 443-451.
27. Iversen, H. H., Holmboe, O., & Bjertnæs, Ø. A. (2012). The Cancer Patient Experiences Questionnaire (CPEQ): reliability and construct validity following a national survey to assess hospital cancer care from the patient perspective. BMJ open, 2(5).
28. Jenkinson, C., Coulter, A., & Bruster, S. (2002). The Picker Patient Experience Questionnaire: development and validation using data from in-patient surveys in five countries. International Journal for Quality in Health Care, 14(5), 353-358.
29. Kriston, L., Scholl, I., Hölzel, L., Simon, D., Loh, A., & Härter, M. (2010). The 9-item Shared Decision Making Questionnaire (SDM-Q-9). Development and psychometric properties in a primary care sample. Patient education and counseling, 80(1), 94-99.
30. Larsson, B. W., & Larsson, G. (2002). Development of a short form of the Quality from the Patient's Perspective (QPP) questionnaire. Journal of clinical nursing, 11(5), 681-687.
31. Lerman, C. E., Brody, D. S., Caputo, G. C., Smith, D. G., Lazaro, C. G., & Wolfson, H. G. (1990). Patients’ perceived involvement in care scale. Journal of general internal medicine, 5(1), 29-33.
32. Lloyd, H., Fosh, B., Whalley, B., Byng, R., & Close, J. (2019). Validation of the person-centred coordinated care experience questionnaire (P3CEQ). International Journal for Quality in Health Care, 31(7), 506-512.
33. Manga, N., Harding, R., De Sa, A., Murie, K., Namane, M. K., Raubenheimer, P. J., ... & De Vries, E. (2018). Development and validation of a tool to measure patient experience in chronic disease care. African journal of primary health care & family medicine, 10(1), 1-7.
34. Mercer, S. W., Maxwell, M., Heaney, D., & Watt, G. (2004). The consultation and relational empathy (CARE) measure: development and preliminary validation and reliability of an empathy-based consultation process measure. Family practice, 21(6), 699-705.
35. O'Cathain, A., Knowles, E., & Nicholl, J. (2011). Measuring patients' experiences and views of the emergency and urgent care system: psychometric testing of the urgent care system questionnaire. BMJ Quality & Safety, 20(2), 134-140.
36. Oltedal, S., Garratt, A., Bjertnæs, Ø., Bjørnsdottìr, M., Freil, M., & Sachs, M. (2007). The NORPEQ patient experiences questionnaire: data quality, internal consistency and validity following a Norwegian inpatient survey. Scandinavian Journal of Public Health, 35(5), 540-547.
37. Parry, C., Mahoney, E., Chalmers, S. A., & Coleman, E. A. (2008). Assessing the quality of transitional care: further applications of the care transitions measure. Medical care, 317-322.
38. Poelstra, R., Selles, R. W., Slijper, H. P., van der Oest, M. J., Feitz, R., Hovius, S. E., ... & Hand-Wrist Study Group. (2018). Better patients’ treatment experiences are associated with better postoperative results in Dupuytren’s disease. Journal of Hand Surgery (European Volume), 43(8), 848-854.
39. Sjetne, I. S., Bjertnaes, O. A., Olsen, R. V., Iversen, H. H., & Bukholm, G. (2011). The Generic Short Patient Experiences Questionnaire (GS-PEQ): identification of core items from a survey in Norway. BMC health services research, 11(1), 1-11.
40. Tremblay, D., Roberge, D., & Berbiche, D. (2015). Determinants of patient-reported experience of cancer services responsiveness. BMC health services research, 15(1), 1-10.
41. Tsui, J. J., Davey, V., & Colvin, L. (2018). The impact of relocation of chronic pain service from hospital setting to community centre on patient experience: a single-centre audit. British journal of pain, 12(4), 220-229.
42. Webster, T. R., Mantopoulos, J., Jackson, E., Cole-Lewis, H., Kidane, L., Kebede, S., ... & Bradley, E. H. (2011). A brief questionnaire for assessing patient healthcare experiences in low-income settings. International Journal for Quality in Health Care, 23(3), 258-268. *[two measures]*
43. Weisse Liste. Methodendokument Versichertenbefragung mit dem Patients‘ Experience Questionnaire (PEQ)
44. Wood, R., Paoli, C. J., Hays, R. D., Taylor-Stokes, G., Piercy, J., & Gitlin, M. (2014). Evaluation of the consumer assessment of healthcare providers and systems in-center hemodialysis survey. Clinical Journal of the American Society of Nephrology, 9(6), 1099-1108.
45. Zinckernagel, L., Schneekloth, N., Zwisler, A. D. O., Ersbøll, A. K., Rod, M. H., Jensen, P. D., ... & Holmberg, T. (2017). How to measure experiences of healthcare quality in Denmark among patients with heart disease? the development and psychometric evaluation of a patient-reported instrument. BMJ open, 7(10).
46. Zlateva, I., Anderson, D., Coman, E., Khatri, K., Tian, T., & Fifield, J. (2015). Development and validation of the Medical Home Care Coordination Survey for assessing care coordination in the primary care setting from the patient and provider perspectives. BMC health services research, 15(1), 1-11.
