# Supplementary material for: Development and content validity of the Experienced Patient‐Centeredness Questionnaire (EPAT)—A best practice example for generating patient‐reported measures from qualitative data
Source: Health Expect. 2022 Apr 21;25(4):1529–38. doi: 10.1111/hex.13494 (PMC9327838; doi:10.1111/hex.13494)
Supplement: Supplementary file 6 — Supporting information. [file HEX-25--s006.docx]

**Appendix 6: Coding tree from qualitative content analysis**

| Dimension | Subcode | Derived from... |
| --- | --- | --- |
| Appropriate communication | General | Model |
|  | Supportive | Model |
|  | Easily understandable language | Model |
|  | Asking questions | Model |
|  | Attention | Model |
|  | Eye contact | Model |
|  | Allowing to finish speaking | Focus group 4 |
|  | Space for reflection | Key informant interview 2 |
|  | Facial expression and gestures | Key informant interview 3 |
|  | Paraphrasing by HCPs | Key informant interview 5 |
|  | Body language perception | Key informant interview 7 |
|  | Paraphrasing by patients | Key informant interview 7 |
|  | Adressing directly | Key informant interview 8 |
|  | Intonation | Key informant interview 8 |
|  | Listening attentively | Measures |
| Consideration of personal circumstances | General | Model |
|  | Holistic view | Model |
|  | Integration body | Model |
|  | Integration mind / soul | Model |
|  | Integration partnership / relationship | Model |
|  | Integration of family and friends | Model |
|  | Integration of work and leisure | Model |
|  | Integration of social origin/living space | Key informant interview 3 |
|  | Integration education | Key informant interview 3 |
| Involvement of family and friends | General | Model |
|  | Preference-based | Model |
|  | Giving information | Model |
|  | Participating in consultations | Model |
|  | Supporting treatment | Model |
|  | Integration of family values | Key informant interview 7 |
|  | Taking care of relatives | Measures |
| Uniqueness of each patient | General | Model |
|  | Needs | Model |
|  | Wishes | Model |
|  | Worries | Model |
|  | Expectations | Model |
|  | Recognizing uniqueness | Model |
|  | Recognizing exceptional situation | Key informant interview 5 |
|  | Subjective understanding of disease | Key informant interview 9 |
| Empowerment of patients | General | Model |
|  | Requirements of everyday life | Model |
|  | Being active in consultations | Model |
|  | Navigating the healthcare systen | Model |
|  | Improving situation | Model |
|  | Access to own data | Focus group 3 |
|  | Self-reliant gathering of information | Focus group 6 |
|  | Competencies of the patient | Key informant interview 4 |
|  | Being active in carrying out the treatment | Key informant interview 4 |
| Integration of additional healthcare elements | General | Model |
|  | Integration on request | Model |
|  | Self-help groups | Model |
|  | Alternative medicine | Model |
|  | Spiritual/pastoral care | Model |
|  | Internet/e-health offers | Key informant interview 10 |
|  | Active exchange regarding offers | Key informant interview 10 |
|  | Information from the HCPs | Key informant interview 3 |
|  | Further training | Key informant interview 8 |
| Good planning of care | General | Model |
|  | Continuity of treatment | Model |
|  | Arrangement of successive treatments | Model |
|  | Appointments for prevention/follow-up care | Model |
|  | Handover/discharge | Model |
|  | Coordination between HCPs | Model |
|  | Fixed contact person | Model |
|  | Arrangements with patient | Key informant interview 1 |
|  | Transparent waiting times | Key informant interview 3 |
|  | Agreements on aims | Key informant interview 8 |
|  | Daily schedule | Measures |
|  | Timely help | Measures |
| Support of physical well-being | General | Model |
|  | Pain management | Model |
|  | Nutrition | Model |
|  | Support everyday life (nursing service) | Model |
|  | Support everyday life (aids) | Model |
| PC characteristics of healthcare providers | General | Model |
|  | Empathy | Model |
|  | Honesty | Model |
|  | Appreciation | Model |
|  | Trustworthiness | Model |
|  | Self-reflection | Model |
|  | Competence | Model |
|  | Taking seriously | Focus group 1 |
|  | Commitment | Focus group 3 |
|  | Openness | Key informant interview 2 |
|  | Respect | Key informant interview 4 |
|  | Introducing oneself | Measures |
| Patient safety | General | Model |
|  | Hygiene | Model |
|  | Evidence-based medicine | Model |
|  | Drug interactions | Model |
|  | Recording treatment progress | Model |
|  | Side effects | Focus group 2 |
|  | Patients' rights | Focus group 3 |
|  | Error management | Focus group 4 |
|  | Contraindications | Focus group 5 |
|  | Data protection | Key informant interview 3 |
|  | Structured processes | Key informant interview 3 |
|  | Safety culture | Key informant interview 4 |
|  | Declaration of consent | Measures |
| Personally tailored information | General | Model |
|  | Information on preventive measures | Model |
|  | Information on disease | Model |
|  | Information on treatments | Model |
|  | Tailored | Model |
|  | Advantages and disadvantages | Model |
|  | Costs | Model |
|  | Patients can share knowledge | Model |
|  | Medication plan | Model |
|  | Room for questions | Focus group 1 |
|  | Use of aids | Key informant interview 10 |
|  | Information on follow-up care | Measures |
| Support of mental well-being | General | Model |
|  | Addressing emotional state | Model |
|  | Psychological offers | Model |
|  | Support in everyday life | Key informant interview 2 |
|  | Activating resources | Key informant interview 7 |
| Collaboration as equal partners and involvement in decision-making | General | Model |
|  | Equality | Model |
|  | Shared decision | Model |
|  | Decision Preference | Model |
|  | Active role of patient | Model |
|  | Invitation to shared-decision making | Key informant interview 6 |
|  | Shared understanding of the problem | Key informant interview 7 |
| Trustful relationship | General | Model |
|  | Constant contact persons | Model |
|  | HCPs informing themselves | Model |
|  | Addressing complaints | Model |
|  | Being able to confide | Focus group 5 |
|  | Forming the relationship | Key informant interview 3 |
|  | Reliability | Key informant interview 5 |
|  | Availability | Key informant interview 7 |
|  | Partner-like relationship | Key informant interview 8 |
| Access to care | General | Model |
|  | Close to home | Model |
|  | Accessible (disability) | Model |
|  | Accessible (language) | Model |
|  | Accessible (other) | Model |
|  | Low payments | Model |
|  | Getting appointment in time | Model |
|  | Flexible appointment making | Model |
|  | Sufficient length of appointments | Model |
|  | Emergency treatment | Model |
|  | Information on specializations/services | Model |
|  | Waiting time | Focus group 1 |
|  | Two-class medicine | Focus group 1 |
|  | Preferential access through contacts | Focus group 6 |
|  | Free choice of treatment | Key informant interview 10 |
|  | Clear signposting | Key informant interview 8 |
|  | Accessible (health literacy) | Key informant interview 8 |
|  | Evaluations of HCPs | Key informant interview 8 |
|  | Procedure of hospital admission | Measures |
|  | Access to aids/tools | Measures |
| Teamwork of healthcare providers | General | Model |
|  | Importance of collaboration | Model |
|  | Interdisciplinarity | Model |
|  | Good arrangements | Model |
|  | Respect | Model |
|  | Trust | Model |
|  | Shared responsibility | Model |
|  | Support by management level | Model |
|  | Common language | Key informant interview 6 |
|  | Super-/Intervision | Key informant interview 6 |
|  | Team spirit | Key informant interview 8 |
|  | Person in charge | Measures |
| HCPs = Health-care professionals | | |
