# Supplementary material for: Development and content validity of the Experienced Patient‐Centeredness Questionnaire (EPAT)—A best practice example for generating patient‐reported measures from qualitative data
Source: Health Expect. 2022 Apr 21;25(4):1529–38. doi: 10.1111/hex.13494 (PMC9327838; doi:10.1111/hex.13494)
Supplement: Supplementary file 7 — Supporting information. [file HEX-25--s002.docx]

**Appendix 7: Examples for item development and first item pool**

**Examples for item development**

Below you can find text examples from the focus groups or key informant interviews for each dimension of patient-centeredness translated into English (original language was German). The examples are followed by the subcodes of the corresponding dimension that we assigned to it and the items that we derived from it. At the end you find a full list of all derived items in German.

**Dimension: Appropriate communication**

Example: *„But generally looking each other in the eye, saying hello, that kind of thing, and also letting people finish speaking and being able to finish the thought, that kind of a thing.“*

(Focus group 4, participant 1)

Subcodes:

- General (Appropriate communication)
- Attention
- Eye contact

Items:

- The HCPs made eye contact with you.
- The HCPs greeted you.
- The HCPs let you finish speaking.

**Dimension: Consideration of personal circumstances**

Example: “*You would have to know, ideally, how he is socialized, what is his profession, what environment does he live in. Does he speak my language, do I have a colleague sitting in front of me that I treat as a patient?*"

(Key informant interview 7)

Subcodes:

- Consideration social origin/living environment
- Consideration profession/leisure time

Items:

- The HCPs took into account whether you speak the national language.
- The HCPs took a holistic view of you.

**Dimension: Involvement of family and friends**

Example: *“There are - I say, the two extremes. The family stays completely out of it. Unfortunately, this often happens, tragically. The other extreme, the family takes over everything. So up to if- I say, if the patient is cared for at home up to taking over the home care. I generally advise against that. Simply because I have seen too often that there, perhaps out of a sense of guilt, an overburdening situation arises, which leads to tension, which leads to an overburdening, of the relatives, to feelings of guilt on the part of the patient and the relatives. And that this is extremely disadvantageous in palliative situations.”*

(Key informant interview 7)

Subcodes:

- Supporting treatment

Items:

- The HCPs asked your relatives if they want to be involved in your treatment.
- The HCPs made it possible for relatives to support you in your treatment.
- The HCPs tried to address your relatives’ feelings.

**Dimension: Uniqueness of each patient**

Example: *“Yes, so I wanted so badly to sleep at home. I would have been hospitalized to get quick help. But I said I was in good hands at home and I had a son who was still 16 at the time, so I wanted to stay there. And it was good for me because I had structure again. I had to come there every day at half past eight, but I was still at home in the evening and could be with my family, with my boyfriend. That was important to me.“*

(Focus group 2 - participant 3)

Subcodes:

- Wishes

Items:

- The HCPs asked about your needs and wishes and took them into consideration.
- The HCPs understood your current situation.
- The HCPs took into account what is particularly important to you.

**Dimension: Empowerment of patients**

Example: “*So and then he saw that I was- also doing a lot myself. We got rid of that. And he said, "Don't do a surgery, it can come back. You can still get that again" and with this sport, so what I do now.”*

(Focus group 4 - participant 6)

Subcodes:

- Improving situation

Items:

- The HCPs encouraged you and were optimistic.
- Were you able to get involved in the treatment?

**Dimension: Integration of additional healthcare elements**

Example: *“And maybe that the same from- from the KISS information service. Maybe from that one first, if it's about cancer. But well, generally just the two, three, so those are for me the two most important sources for health information. And that- that would also be a sign, which one sets. And then maybe also on, I don't know, NAKOS or whoever mediates self-help groups, addresses and simply just their openness, willingness.“*

(Key informant interview 6)

Subcodes:

- Information from the HCPs

Items:

- The HCPs informed you about self-help groups.
- The HCPs gave you specific addresses or telephone numbers where you can get further training.

**Dimension: Good planning of care**

Example: *“Or pass it on to the respective colleagues. But that would also be part of it for me, yes. So you also have to organize the division of labor, and let's take the topic of anesthesia, it is always the case in a surgery that you simply say: "There is now another explanation of the anesthesia.” And that this builds on each other, so that the explanation of the anesthesia naturally builds on what the surgeon has perhaps done to explain the surgical treatment.”*

(Key informant interview 3)

Subcodes:

- Coordination between HCPs
- Arrangement of successive treatments

Items:

- Your different HCPs were coordinated with each other.
- If multiple practitioners educated you, the conversations built logically on each other.

**Dimension: Support of physical well-being**

Example: *“So that they actually tried to help me immediately because I had very, very severe pain.”*

(Focus group 4 - participant 6)

Subcodes:

- Pain management

Items:

- The people treating you made an effort to treat your pain.

**Dimension: PC characteristics of healthcare providers**

Example: “*Yes, so I can quite simply say what you also meant, talking to each other at eye level, in other words, not being pigeonholed.”*

(Focus group 4 - participant 1)

Subcodes:

- Appreciation

Items:

- The HCPs patronized you.
- The HCPs pigeonholed you without listening to you.

**Dimension: Patient safety**

Example: *“Are we doing the right intervention? Are the right people there? Do we have the instruments here? Ultimately, all of the things that are attempted via such "patient safety" campaigns. Then again, explicitly demanding attention, patient checklists and all these intermediate steps.”*

(Key informant interview 10)

Subcodes:

- Safety culture
- Structured processes

Items:

- The HCPs adhered to a culture of safety.
- The HCPs used tools such as checklists to ensure that all important steps have occurred.

**Dimension: Personally tailored information**

Example: *“Are they put down for you and do they say: "Here, swallow this" or are you told: "So, starting tomorrow, a new medication will be put into this bag, you will get it because of that and look, pay attention, this could do this and that" or something like that.”*

(Key informant interview 1)

Subcodes:

- Information on treatments

Items:

- The HCPs explained clearly why you are being given this medication, how it works, how you should take it, and how it interacts with other medications.
- The HCPs educated you in detail about the treatment.

**Dimension: Support of mental well-being**

Example: *“…they always come as well. No, I've had good experience with them. When I was in the hospital at that time, a green lady* (note: a volunteer mental health service) *came and I was able to lay down all my problems and tears with her. And that felt very good to me after the surgery and so on.”*

(Focus group 1 - participant 2)

Subcodes:

- Psychological offers

Items:

- The HCPs have been responsive to your fears and concerns.
- The HCPs made an effort to make you feel better.
- The HCPs supported you in coping with your illness.

**Dimension: Collaboration as equal partners and involvement in decision-making**

Example: *“And at the same time also say that I'm not taking the decision away from you. It's your decision, but also to convey to the patient, "Whatever decision you make, I'll go along with it." I experience quite often that physicians are offended because a patient makes a different decision than the recommendation.”*

(Key informant interview 9)

Subcodes:

- Decision preference
- Shared decision

Items:

- The HCPs left the treatment decision up to you.
- The HCPs respected your decision, even if it was different from the HCP’s recommendation.

**Dimension: Trustful relationship**

Example: *“I really feel like I'm in good hands there. Because he, yes, he knows me by now. I have a tear running down my face, he knows what's bothering me emotionally, and you have to wait longer, but I and many patients are happy to put up with that because you can really confide in him.”*

(Focus group 5 - participant 2)

Subcodes:

- Being able to confide

Items:

- You felt like that you were in good hands.
- The HCPs knew what was bothering you emotionally.

**Dimension: Access to care**

Example: *“Why doesn't anyone tell me how long I have to wait and who I actually have to see.”*

(Key informant interview 8)

Subcodes:

- Waiting time

Items:

- The HCPs informed you about how long you have to wait.
- You have been told which HCPs you are waiting for.

**Dimension: Teamwork of healthcare providers**

Example: *“So this aspect, like super- and intervision, that you can say, you then ultimately follow together a shared policy through the shared agreements, because we have so far only the good and shared agreements, but not this shared policy and that all treat the patient the same.”*

(Key informant interview 6)

Subcodes:

- Super-/Intervision

Items:

- The team's HCPs pursued common goals.
- There is supervision/intervision for the HCP team.

**First item pool**

Below are all items of the first item pool in German. Please note, that for most items there was also a memo which included alternative wordings, context information or other comments by the coders. There are many items with similar wordings. This happened as some quotes from the qualitative sources where included in more than one dimension. For example, a quote about how the HCPs should give information about the treatment in an understandable way would have been coded with the dimension “Appropriate communication” as well as the dimension “Personally tailored information”. Thus, this quote would have been coded with items twice (once for each dimension) leading to similar items. Those double items were deleted afterwards in team discussions.

| Dimension: Angemessene Kommunikation |
| --- |
| Wie gut konnten Sie die Erklärungen der Behandelnden verstehen? |
| Die Behandelnden haben Blickkontakt zu Ihnen aufgebaut. |
| Die Behandelnden haben nicht drumherum geredet. |
| Die Behandelnden haben ihre Sprache so angepasst, dass Sie sie gut verstehen konnten. |
| Die Behandelnden haben eine Sprache verwendet, die Sie trotz Barrieren (Fremdsprache, Behinderung, akuter Zustand) gut verstehen konnten. |
| Die Behandelnden haben Ihre Fragen ernst genommen und freundlich beantwortet. |
| Die Behandelnden haben über Sie hinweg gesprochen. |
| Die Behandelnden haben Ihnen das Gefühl gegeben, Fragen stellen zu können. |
| Die Behandelnden haben Sie kompetent behandelt. |
| Die Behandelnden haben wirkten interessiert. |
| Die Behandelnden haben eine aufgeschlossene Körperhaltung gezeigt. |
| Die Behandelnden haben Ihre Wünsche ernst genommen. |
| Die Behandelnden haben Besprochenes wiederholt um sicherzustellen, dass sie Sie richtig verstanden haben. |
| Die Behandelnden haben sich auf Sie konzentriert. |
| Die Behandelnden haben die Behandlung mit Ihnen gemeinsam besprochen. |
| Die Behandelnden haben mit Smalltalk oder Humor eine entspannte Stimmung aufgebaut. |
| Die Behandelnden haben Sie begrüßt. |
| Die Behandelnden haben wichtige Fragen zu Ihrem Gesundheitsproblem gestellt. |
| Die Behandelnden haben Sie mit einbezogen in die Behandlungsentscheidung. |
| Die Behandelnden haben auf Augenhöhe mit Ihnen gesprochen. |
| Die Behandelnden haben Ihnen alle relevanten Informationen gegeben. |
| Die Behandelnden haben mit Ihnen in einer ruhigen Stimme gesprochen. |
| Die Behandelnden haben eine für Sie verständliche Sprache verwendet. |
| Die Behandelnden haben eine angemessene Sprache verwendet. |
| Die Behandelnden haben Zusammenhänge gut erklärt. |
| Die Behandelnden haben eine vertrauensvolle Beziehung zu Ihnen aufgebaut. |
| Wie leicht fiel es Ihnen mit den Behandelnden zu sprechen? |
| Die Behandelnden haben auch nonverbale Signale wahrgenommen. |
| Die Behandelnden haben sich ausreichend Zeit genommen damit Sie alles verstehen konnten. |
| Die Behandelnden haben Ihnen ausreichend Zeit gegeben Fragen zu beantworten. |
| Die Behandelnden haben Sie ermutigt. |
| Die Behandelnden haben Ihnen Raum gegeben Ihre Vorstellungen zu beschreiben. |
| Die Behandelnden haben sichergestellt, dass Sie trotz Barrieren gut miteinander kommunizieren konnten. |
| Die Behandelnden haben Sie ernst genommen. |
| Die Behandelnden sind darauf eingegangen, was die Situation für Sie persönlich bedeutet. |
| Die Behandelnden waren ausgeglichen im Gespräch mit Ihnen. |
| Die Behandelnden haben Sie ermutigt Ihre eigene Meinung zu Ihrer Behandlung zu teilen. |
| Die Behandelnden haben Sie ermutigt, über Ihre eigenen Sorgen zu sprechen |
| Die Behandelnden haben Grafiken oder Dokumente genutzt um ihre Erklärungen zu unterstützen. |
| Die Behandelnden haben ausreichend mit Ihnen gesprochen. |
| Die Behandelnden haben sichergestellt, dass Sie alles richtig verstanden haben. |
| Die Behandelnden haben versucht Ihnen etwas zu verbieten. |
| Die Behandelnden haben Verständnis dafür, wenn Sie distanziert erscheinen. |
| Die Behandelnden haben sich klar ausgedrückt. |
| Die Behandelnden haben berücksichtigt, wie Sie informiert werden wollten. |
| Die Behandelnden haben sich bei Ihnen entschuldigt, wenn Fehler gemacht wurden. |
| Die Behandelnden haben Sie zu Beginn begrüßt. |
| Die Behandelnden haben Sie unterstützt Lösungen zu finden. |
| Die Behandelnden haben Ruhe ausgestrahlt. |
| Die Behandelnden haben Sie zu Ihren Themen gut beraten. |
| Die Behandelnden haben Sie in positiv bestärkt. |
| Die Behandelnden haben die Behandlungsoptionen mit Vor- und Nachteilen besprochen. |
| Die Behandelnden haben waren ehrlich zu Ihnen. |
| Die Behandelnden haben Sie ausreichend untersucht. |
| Die Behandelnden haben sich Zeit für Sie genommen. |
| Die Behandelnden haben Ihre Sorgen und Beschwerden ernst genommen. |
| Die Behandelnden haben Sie als einzigartigen Menschen behandelt. |
| Die Behandelnden waren einfühlsam. |
| Die Behandelnden waren engagiert. |
| Die Behandelnden haben sich vorgestellt. |
| Die Behandelnden haben Ihnen aufmerksam zugehört. |
| Die Behandelnden haben Sie ausreden lassen. |

| Dimension: Berücksichtigung Lebensumstände |
| --- |
| Die Behandelnden haben auch mögliche private Themen angesprochen. |
| Die Behandelnden haben Sie nicht nur als Hülle, sondern als Mensch gesehen. |
| Die Behandelnden haben Ihre Persönlichkeit bei der Behandlung beachtet. |
| Die Behandelnden haben Ihre Lebensgeschichte berücksichtigt. |
| Die Behandelnden haben bei der Planung Ihrer Behandlung Ihre persönlichen Umstände und Wünsche berücksichtigt. |
| Die Behandelnden haben Sie darüber aufgeklärt, wann Sie wieder alltäglichen Tätigkeiten nachgehen können. |
| Die Behandelnden haben Sie ganzheitlich betrachtet. |
| Die Behandelnden haben berücksichtigt, ob Sie in der Lage sind zur Behandlung zu kommen. |
| Die Behandelnden haben berücksichtigt, ob Sie sich selbst zuhause versorgen können. |
| Die Behandelnden haben Ihre familiäre Situation berücksichtigt. |
| Die Behandelnden haben berücksichtigt, wie gut Sie sozial eingebunden sind. |
| Die Behandelnden haben berücksichtigt, ob Sie körperlich fit genug für die Behandlung waren. |
| Die Behandelnden haben sich Ihrem Sprachniveau angepasst. |
| Die Behandelnden haben überprüft, ob Sie möchten, dass Ihre Familie eingebunden wird. |
| Die Behandelnden haben dabei geholfen, dass Ihre sozialen Kontakte sich verbessert haben. |
| Die Behandelnden haben berücksichtigt, dass bei einer körperlichen Erkrankung auch die Psyche beteiligt sein kann. |
| Die Behandelnden haben Beziehungsthemen in Bezug zu Ihrer Erkrankung angesprochen und Unterstützung angeboten. |
| Die Behandelnden haben Ihre Wünsche nach zusätzlichen Behandlungsangeboten berücksichtigt. |
| Die Behandelnden haben erfragt, wie meine Krankheit Ihr Leben beeinflusst. |
| Die Behandelnden haben Ihre körperlichen Beschwerden nicht auf die Psyche reduziert. |
| Die Behandelnden haben Ihr Alter berücksichtigt. |
| Die Behandelnden haben Sie individuell behandelt. |
| Die Behandelnden haben Ihre Interessen berücksichtigt. |
| Die Behandelnden haben Rücksicht auf Ihre Ängste genommen. |
| Die Behandelnden haben berücksichtigt, ob Sie geistig fit genug sind. |
| Die Behandelnden haben die mögliche Belastung Ihrer Angehörigen berücksichtigt. |
| Die Behandelnden haben alle Bereiche Ihres Körpers bei der Behandlung beachtet. |
| Die Behandelnden haben ihre berichteten Probleme ernst genommen. |
| Die Behandelnden haben Ihre körperlichen Beschwerden berücksichtigt. |
| Die Behandelnden haben leicht verständlich gesprochen, damit Sie alles verstehen |
| Die Behandelnden haben berücksichtigt, ob Sie die Landessprache sprechen. |
| Die Behandelnden haben Ihre psychischen Erkrankungen berücksichtigt. |
| Die Behandelnden haben Ihre Pflichten zuhause oder bei der Arbeit berücksichtigt |
| Die Behandelnden haben berücksichtigt, ob Sie soziale Unterstützung erhalten. |
| Die Behandelnden haben auch andere Fachbereiche bei der Behandlung betrachtet. |
| Die Behandelnden haben Sie bei der Problemlösung unterstützt. |

| Einbezug Familie und Freunde |
| --- |
| Die Behandelnden haben Ihre Angehörigen (Familie, Freunde) mit in die Behandlung einbezogen. |
| Die Behandelnden haben Ihre Angehörigen gefragt, ob sie in Ihre Behandlung miteinbezogen werden wollen. |
| Die Behandelnden haben es ermöglicht, dass Ihre Angehörigen Sie bei der Behandlung unterstützen konnten. |
| Die Behandelnden haben versucht auf die Befindlichkeiten Ihrer Angehörigen einzugehen. |
| Die Behandelnden haben Ihre Angehörigen mit Informationen über Erkrankung versorgt. |
| Die Behandelnden haben Ihren Wunsch Angehörige einzubeziehen berücksichtigt. |
| Wie sehr wurden Sie als Eltern von den Behandelnden in die Behandlung Ihres Kindes miteinbezogen. |
| Wie sehr wurden Sie von den Behandelnden unterstützt Ihre eigenen Werte und die Ihrer Familie in der Behandlung zu berücksichtigen? |
| Wie sehr hat Ihr Kind die Behandlung erhalten, welche Sie sich gewünscht haben? |
| Wie sehr wurden die Wünsche und Bedürfnisse Ihrer Angehörigen in Behandlungsentscheidungen mit einbezogen? |
| Die Behandelnden haben Ihre Angehörigen über zusätzliche Hilfsangebote informiert. |
| Die Behandelnden haben sich bemüht Ihnen die Behandlung Ihres Kindes zu erklären. |
| Die Behandelnden haben die Einwände Ihrer Angehörigen ernst genommen. |
| Die Behandelnden haben gegenüber Ihren Angehörigen Ihre Privatsphäre geschützt. |
| Wie sehr waren die Behandelnden bemüht bei möglichen Diskrepanzen bezüglich der Behandlung zwischen Ihnen und Ihren Angehörigen zu vermitteln? |
| Die Behandelnden haben Sie über die Möglichkeit Ihre Angehörigen mit einzubeziehen informiert. |
| Die Behandelnden haben im Anschluss an ein Gespräch gemeinsam mit Ihren Angehörigen ein weiteres Gespräch mit Ihnen alleine geführt. |
| Wie sehr wurde Ihren Angehörigen von den Behandelnden die Möglichkeit gegeben sich z.B. in einem Gespräch an der Behandlung zu beteiligen? |

| Dimension: Einzigartigkeit |
| --- |
| Die Behandelnden haben Ihren Zustand berücksichtigt. |
| Die Behandelnden haben Sie als einzelnen Menschen gesehen. |
| Die Behandelnden haben Ihre Sichtweisen erfragt und berücksichtigt. |
| Die Behandelnden haben Ihre Bedürfnisse und Wünsche erfragt und berücksichtigt. |
| Die Behandelnden haben Sie in einer akuten Situation nicht bedrängt. |
| Die Behandelnden haben Ihnen Raum gegeben, Ihre Situation zu schildern. |
| Die Behandelnden haben Ihre aktuelle Situation verstanden. |
| Die Behandelnden haben Ihre Geschlechtsidentität berücksichtigt. |
| Die Behandelnden haben Sie fair behandelt und Sie nicht diskriminiert. |
| Die Behandelnden haben sich in der Gesprächsführung an Sie angepasst. |
| Die Behandelnden haben Sie als Mensch und nicht nur Ihre Krankheit behandelt. |
| Die Behandelnden haben Ihre Werte und Einstellungen berücksichtigt. |
| Die Behandelnden haben bei der Behandlung Ihre Ängste berücksichtigt. |
| Die Behandelnden haben Ihnen altersgerecht vermittelt. |
| Wie sehr waren die Behandelnden aufmerksam? |
| Wie lange mussten Sie in der Praxis/Krankenhaus warten? |
| Die Behandelnden haben eine persönliche und vertrauensvolle Beziehung aufgebaut. |
| Die Behandelnden haben anerkannt, dass die Situation eine Ausnahmesituation für Sie darstellt. |
| Die Behandelnden haben Ihre Ressourcen berücksichtigt. |
| Die Behandelnden haben Ihr Alter berücksichtigt. |
| Die Behandelnden haben, wenn gewünscht, eine Begleitung sichergestellt. |
| Die Behandelnden haben Ihnen schriftliche Informationen zu Ihrer Behandlung zur Verfügung gestellt. |
| Die Behandelnden haben eine vertrauenswürdige Beziehung aufgebaut. |
| Hatten Sie die Möglichkeit die Behandelnden bei Probleme zu wechseln? |
| Die Behandelnden haben versucht Ihnen Zusatzleistungen ohne Indikation anzubieten. |
| Die Behandelnden haben mit Ihnen besprochen wie Sie Ihre persönlichen Ziele erreichen können. |
| Die Behandelnden haben Sie so gründlich wie gewünscht untersucht. |
| Die Behandelnden haben die Therapie auf Sie individuell zugeschnitten. |
| Die Behandelnden haben berücksichtigt, was Ihnen besonders wichtig ist. |
| Die Behandelnden haben Ihre Kultur und Religion berücksichtigt. |
| Die Behandelnden haben erfragt, was Ihnen in der Behandlung geholfen hat. |
| Die Behandelnden haben gefragt, was Sie glauben, was Ihre Symptome auslöst. |
| Wie sehr mussten Sie Druck ausüben, damit Sie unterstützt wurden? |
| Die Behandelnden haben Ihre Erwartungen berücksichtigt. |
| Die Behandelnden haben Sie als Experten für Ihre Erkrankung und Behandlung einbezogen |
| Die Behandelnden haben ihre Ziele bei der Behandlung berücksichtigt. |
| Die Behandelnden haben Sie über verschiedene Behandlungsmöglichkeiten aufgeklärt. |
| Die Behandelnden haben Sie über die Behandlungskosten aufgeklärt. |
| Die Behandelnden haben ehrlich mit Ihnen kommuniziert. |
| Die Behandelnden haben nicht über Sie hinweg gesprochen. |
| Die Behandelnden haben Sie und Ihre Beschwerden ernst genommen. |
| Die Behandelnden haben Sie kompetent behandelt. |
| Die Behandelnden haben Ihnen die Informationen gegeben, die Sie wollten. |
| Die Behandelnden haben auf Wunsch Ihre Angehörigen mit einbezogen. |
| Die Behandelnden haben Sie in die Behandlungsentscheidung mit einbezogen. |
| Die Behandelnden haben Ihre Schmerzen gelindert. |
| Wie gut/schnell war der Zugang zur einer Notfall Versorgung? |
| Die Behandelnden haben Ihren beruflichen Kontext berücksichtigt. |
| Die Behandelnden haben Sie respektvoll behandelt. |
| Die Behandelnden haben Sie auf Augenhöhe behandelt. |
| Die Behandelnden waren engagiert. |
| Die Behandelnden haben sich empathisch und einfühlsam verhalten. |
| Die Behandelnden haben sich genug Zeit für Sie genommen. |
| Die Behandelnden haben Sie in Ihre Versorgung eingebunden. |
| Die Behandelnden haben Sie als Ganzes gesehen. |
| Die Behandelnden haben Sie ohne Vorurteile behandelt. |
| Die Behandelnden haben auf Ihre Kritik reagiert. |
| Die Behandelnden haben Ihre Sorgen und Bedenken berücksichtigt. |
| Die Behandelnden haben verschiedene Bereiche Ihres Lebens berücksichtigt. |

| Dimension: Empowerment |
| --- |
| Haben Sie selbst Medikamente abgesetzt? |
| Konnten Sie sich in der Behandlung einbringen? |
| Sie konnten erklären, was Sie sich wünschen in der Behandlung? |
| Die Behandelnden haben Sie unterschätzt. |
| Sie konnten einen Behandlungsplan mitbestimmen. |
| Die Behandelnden haben Ihnen bei der Zielsetzung geholfen. |
| Sie konnten mitentscheiden, obwohl die Verantwortung bei den Behandelnden lag. |
| Sie waren Teil Ihres eigenen Behandlungsteams. |
| Sie wussten, wie Sie Ihre eigene Gesundheit verbessern konnten. |
| Die Behandelnden haben Ihnen klar vermittelt, was Sie tun müssen, um Ihre Gesundheit zu verbessern. |
| Die Behandelnden haben Sie bestärkt und waren optimistisch. |
| Haben Sie sich in einer Selbsthilfegruppe austauschen können? |
| Haben Sie sich selbst informiert vor dem Termin? |
| Konnten Sie sich selbst Informationen beschaffen? |
| Die Behandelnden haben Ihnen Tipps gegeben und dadurch geholfen. |
| Die Behandelnden haben Ihnen etwas versprochen und es nicht erfüllt. |
| Die Behandelnden haben vermittelt, dass Sie trotz Ihrer Krankheit immer noch ein Mensch sind und bleiben. |
| Die Behandelnden haben Ihnen erklärt, wie Sie Ihre Behandlung zuhause durchführen können. |
| Die Behandelnden haben erklärt, wann Sie wieder Ihren Alltagsaktivitäten nachkommen können. |
| Die Behandelnden haben Ihnen geholfen für schwierige Situationen in der Zukunft vorbereitet zu sein. |
| Die Behandelnden haben Ihnen aufgezeigt, wie sich Ihr Verhalten auf Ihre Gesundheit auswirkt. |
| Wie schnell konnten Sie in einer akuten Situation Hilfe bekommen? |
| Die Behandelnden haben Ihnen ermöglicht, die Dinge, zu denen Sie im Stande sind, auch selbst zu tun. |
| Die Behandelnden haben Ihre eigene Erfahrung miteinbezogen. |
| Die Behandelnden haben Ihnen erklärt, was Sie tun müssen, um gesund zu bleiben. |
| Die Behandelnden haben sich mit Ihnen auf Augenhöhe unterhalten. |
| Die Behandelnden haben Sie über Prävention aufgeklärt. |
| Wussten Sie, wohin Sie sich wenden konnten, um Leistungen oder Unterstützung zu erhalten? |
| Konnten Sie Fragen stellen? |
| Waren Sie dazu gezwungen, Ihre Pflege zuhause selbst zu organisieren? |
| Die Behandelnden haben Sie bestärkt, Ihr Leben wieder so zu leben, wie es früher war. |
| Haben Sie in Eigenregie alternative Methoden ausprobiert. |
| Die Behandelnden haben Sie gefragt, was Sie selbst tun können, um Ihre Situation zu verbessern. |
| Haben Sie einen Entlassbericht erhalten. |
| Die Behandelnden haben einen Behandlungsplan mit Ihnen besprochen. |
| Die Behandelnden haben Sie darin unterstützt Ihre Gesundheit selbst zu managen. |
| Die Behandelnden haben gegenüber Ihnen Verbote ausgesprochen. |
| Die Behandelnden haben Sie darin unterstützt sich so zu informieren und fortzubilden, dass Sie sich gut in Behandlungsentscheidungen einbringen können. |
| Die Behandelnden haben Sie motiviert Programme in Ihrer Umgebung zu besuchen. |
| Mussten Sie sich gegen Ihre Behandelnden durchsetzen, um das zu erhalten, was Sie Ihrer Meinung nach brauchten? |
| Die Behandelnden waren ehrlich mit Ihnen. |
| Die Behandelnden haben gefragt, ob Sie wissen, wo Sie Informationen zu Ihrer Gesundheit im Internet finden. |
| Die Behandelnden haben Sie motiviert, sich an der Behandlung zu beteiligen. |
| Die Behandelnden haben Ihnen erklärt, wohin Sie sich wenden können wenn Sie außerhalb der Öffnungszeiten einen Arzt brauchen. |
| Die Behandelnden haben Sie dabei unterstützt Formalitäten wie Anträge zu organisieren. |
| Die Behandelnden haben Ihnen eine Liste gegeben, damit Sie wissen, was Sie tun müssen. |
| Die Behandelnden haben Sie ermutigt selbst mitzudenken. |
| Die Behandelnden haben Ihnen genau mitgeteilt welche Medikamente Sie erhalten. |
| Die Behandelnden haben Ihnen schriftliche Informationen gegeben welche Behandlungen durchgeführt wurden. |
| Die Behandelnden haben Ihre Wünsche in der Behandlung berücksichtigt. |
| Die Behandelnden haben Sie motiviert sich aktiv in die Behandlung einzubringen. |
| Die Behandelnden haben Ihre Anregungen zur Behandlung oder Untersuchung umgesetzt. |
| Konnten Sie Ihre Rechte durchsetzen? |
| Die Behandelnden haben die Informationen, die Sie einbringen konnten, genutzt. |
| Die Behandelnden haben Ihnen mit Absicht Dinge nicht erzählt. |
| Die Behandelnden haben Sie motiviert, wieder Hobbies nachzugehen. |
| Die Behandelnden haben Sie alleine gelassen bei der Arztsuche. |
| Die Behandelnden waren daran interessiert, was Sie über Ihre Krankheit wissen. |
| Die Behandelnden haben Sie informiert, wie Sie sich um sich kümmern können. |
| Die Behandelnden sind mehr auf das von Ihnen Gesagte eingegangen. |
| Waren Sie mitverantwortlich für Ihre Behandlung? |
| Die Behandelnden haben Sie aufgeklärt. |
| Die Behandelnden haben auch andere Bereiche in die Behandlung einbezogen. |
| Die Behandelnden haben sich an Absprachen gehalten. |
| Wie sehr mussten Sie selbst Teile der Behandlung anstoßen? |

| Dimension: Ergänzende Angebote |
| --- |
| Wurden die Kosten für alternative Medizin durch die Krankenkasse übernommen? |
| Die Behandelnden haben Ihre Gesundheit ganzheitlich betrachtet. |
| Sie konnten mit verschiedenen Behandelnden sprechen, die verschieden Schwerpunkte hatten, damit die Versorgung Ihren persönlichen Bedürfnissen entspricht. |
| Die Behandelnden haben Sie ermutigt, zusätzliche Angebote zu nutzen (z.B. Selbsthilfegruppen). |
| Die Behandelnden haben Ihnen ergänzende Angebote (z.B. …) angeboten. |
| Die Behandelnden haben Sie darüber aufgeklärt, welche zusätzlichen Leistungen die Praxis/Station anbietet. |
| Die Behandelnden haben Sie zu alternativen Behandlungsmethoden aufgeklärt. |
| Die Behandelnden hatten kompetentes Wissen zu alternativen Behandlungsmethoden. |
| Die Behandelnden haben Ihnen psychologische Unterstützung angeboten. |
| Die Behandelnden haben alternative und schulmedizinische Verfahren gleichberechtigt mit Ihnen besprochen. |
| Die Behandelnden haben interdisziplinär gearbeitet, um Sie ganzheitlich zu behandeln. |
| Die Behandelnden haben Ihnen unterstützende Angebote für organisatorische Aspekte angeboten. |
| Die Behandelnden haben anerkannt, wenn Sie alternative Behandlungsmethoden genutzt haben. |
| Die Behandelnden haben Sie über Selbsthilfegruppen informiert. |
| Die Behandelnden haben waren in der Lage Ihnen auf Nachfrage Informationen zu ergänzenden Angeboten zu geben. |
| Die Behandelnden haben Sie gefragt ob Sie Interesse an alternativen Behandlungsmethoden haben. |
| Die Behandelnden haben alternative Behandlungsmethoden auf Ihren Wunsch mit in die Behandlung einbezogen. |
| Die Behandelnden haben Ihnen Fortbildungen zu Ihrer Erkrankung und Behandlung angeboten. |
| Die Behandelnden haben Ihnen konkrete Adressen oder Telefonnummern gegeben, bei denen Sie sich weiter fortbilden können. |
| Die Behandelnden konnten Ihnen erklären wo Sie vertrauenswürdige Informationen zu Ihrer Erkrankung und Behandlung finden. |
| Die Behandelnden haben, wenn sie sich gegen ergänzende Angebote ausgesprochen haben, erklärt aus welchen Gründen sie dagegen sind. |
| Die Behandelnden haben Sie dabei unterstützt Termine zur Weiterbildung zu Ihrer Erkrankung zu erhalten. |
| Die Behandelnden konnten Ihnen Fragen zu ergänzenden Angeboten beantworten. |
| Die Behandelnden haben hat Sie ermutigt mitzuteilen welche ergänzenden Angebote Sie nutzen. |
| Die Behandelnden haben Ihnen auch emotionale Unterstützung angeboten. |
| Die Behandelnden haben Sie in der Behandlung durch eine Person unterstützt, die Ihnen erklärt hat, wo Sie Hilfe bekommen oder den nächsten Termin haben. |

| Dimension: Gute Planung |
| --- |
| Die Behandelnden haben zeitlich klare Termin mit Ihnen vereinbart und eingehalten. |
| Wenn mehrere Behandler Sie aufgeklärt haben, bauten die Gespräche logisch aufeinander auf. |
| Waren Aufzeichnungen über Ihre Behandlung/Gesundheit für andere Behandler verfügbar, so dass sie die Behandlung gut planen konnten? |
| Die Behandelnden haben Ihnen den Ablauf verschiedener Schritte Ihrer Untersuchung/Behandlung erklärt. |
| Die Behandelnden haben verschiedenen Schritte Ihrer Behandlung gut aufeinander abgestimmt. |
| Wie oft mussten Sie verschiedenen Behandelnden Ihre Gesundheitsprobleme schildern? |
| Nach der Entlassung aus dem Krankenhaus, wusste mein Hausarzt… ... welche Medikamente mir verschrieben wurden/ob sich etwas in der Verschreibung geändert hat. ... wie ich im Krankenhaus behandelt wurde. ... welche Behandlungen/Schritte nach dem Krankenhausaufenthalt anstehen. |
| Die Behandelnden haben Sie dabei unterstützt einen Termin bei einem anderen Behandelnden zu bekommen. |
| Ihre verschiedenen Behandelnden haben sich miteinander abgesprochen. |
| Sie hatten einen Ansprechpartner, der Ihre Versorgung koordiniert hat. |
| Behandler verschiedener Fachrichtungen haben bei Ihrer Versorgung gut zusammengearbeitet. |
| Die Behandelnden haben Ihnen gut erklärt, wie Sie Ihre Behandlung zu Hause selbst umsetzen können. |
| Die Behandelnden haben Ihnen erklärt wie Sie einen Termin bei einem anderen Behandelnden machen. |
| Die Behandelnden haben Absprachen mit Ihnen eingehalten. |
| Die Behandelnden haben Ihnen gut erklärt warum welche Untersuchungen gemacht wurden und was die Untersuchungsergebnisse bedeuten. |
| Die Behandelnden haben Ihnen gut erklärt, auf welche Symptome Sie achten müssen um festzustellen ob sich Ihre Erkrankung verschlimmert oder verbessert. |
| Die Behandelnden haben Ihnen gut erklärt, bei welchen Symptomen Sie sich an welchen Arzt wenden (z.B. wann Notarzt und wann zum Hausarzt etc.). |
| Die Behandelnden haben Ihnen gut erklärt, wie Sie Ihre Medikamente einnehmen sollten, wie sie wirken und wann sie abgesetzt werden sollten. |
| Die Behandelnden haben Ihnen gut erklärt welche Behandlungen warum durchgeführt werden und wie lange die Behandlungen dauern. |
| Waren die Wartezeiten angemessen? |
| Die Behandelnden haben Sie gut auf Ihre Entlassung vorbereitet. |
| Die Behandelnden waren gut darüber informiert, welcher Behandlungsschritt an welchem Punkt im Behandlungsplan Sie gerade sind. |
| Wenn sich Termine oder Absprachen verändert haben, wurde Ihnen das mitgeteilt und ein Grund genannt. |
| Die Behandelnden haben Ihnen erklärt, wann Sie Alltagsaktivitäten wieder aufnehmen können (z.B. Arbeit oder Auto fahren). |
| Die Behandelnden haben Ihnen die weiteren Schritte gut erklärt. |
| Die Behandelnden haben Ihnen mitgeteilt wie lang die Behandlung realistisch dauert. |
| Wenn Sie Hilfe benötigten, erhielten Sie diese schnell z.B. um ins Badezimmer zu gehen. |
| Ihre Versorgung war gut organisiert. |
| Ihre Versorgung hatte eine hohe Qualität (hohe Kompetenz, moderne Behandlungsmethoden). |
| Sie hatten einen Ansprechpartner, der Ihnen geholfen hat sich im Gesundheitssystem zurechtzufinden (z.B. Termine vereinbaren, Formulare ausfüllen, Anträge stellen). |
| Sie hatten die Möglichkeit denselben/dieselbe Behandelnde zu sprechen. |
| Die Behandelnden haben Ihnen erklärt, wie Sie das Personal kontaktieren können. |
| Die Behandelnden haben sichergestellt, dass Ihre Behandlung ausreichend lange durchgeführt wird. |
| Die Behandelnden haben sichergestellt, dass bei Bedarf der nächste Behandlungsschritt direkt anschließt. |
| Die Behandelnden haben Sie nach der Behandlung kontaktiert, um zu erfragen, wie es Ihnen geht. |
| Wenn Sie zwischen Stationen verlegt wurden, war das gut organisiert. |
| Bei Entlassung aus dem Krankenhaus haben Sie einen Entlassbrief für Ihren Hausarzt erhalten. |
| Die Behandelnden haben gemeinsam mit Ihnen realistische Ziele vereinbart und schriftlich festgehalten. |
| Die Behandelnden haben Sie dabei unterstützt Ihre Ziele zu erreichen. |
| Die Behandelnden haben bei Bedarf Ihr Familie oder Freunde mit in die Behandlung einbezogen. |
| Die Behandelnden haben Sie gut durch den Genesungsprozess nach der Entlassung geführt. |
| Die Behandelnden haben Sie gut informiert welches der nächste Behandlungsschritt ist. |
| Sie konnten schnell Antworten bekommen. |
| Die Behandelnden reagierten schnell auf den Rufknopf. |
| Sie haben auch außerhalb der Öffnungszeiten Hilfe erhalten können. |
| Die Behandelnden haben Ihnen mitgeteilt was beim nächsten Termin gemacht wird. |
| Die Behandelnden haben Ihnen die alltägliche Routine erklärt. |
| Die Behandelnden haben Nachsorgetermine mit Ihnen abgesprochen. |
| Die Behandelnden haben am Ende Ihrer Behandlung über die Nachsorge oder weitere Behandlung Ihrer Erkrankung aufgeklärt. |
| Die Behandelnden haben Ihre Nachsorge gut organisiert. |
| Die Behandelnden haben Ihnen mitgeteilt, wie schnell Sie Hilfe für Ihre Gesundheitsproblem benötigen. |
| Die Behandelnden haben mitgeteilt, in welcher Reihenfolge Sie und die anderen Patienten rankommen. |
| Sie haben rechtzeitig einen Termin bekommen. |
| Die Behandelnden haben Ihnen erklärt, wie lange Sie warten müssen. |
| Die Behandelnden haben mit Ihnen besprochen wie Sie Ihre Gesundheitsziele erreichen können. |
| Die Behandelnden haben Sie nach Ihren persönlichen Gesundheitszielen gefragt. |
| Die Behandelnden haben mit Ihnen einen Behandlungsplan für den Alltag erstellt. |
| Wie sehr mussten Sie dafür sorgen, dass Behandelnde die Testergebnisse von anderen Behandelnden erhalten haben. |
| Ihnen wurde erklärt, warum Sie warten müssen. |
| Die Behandelnden haben private Aspekte bei Ihrer Entlassung berücksichtigt. |
| Wie angenehm war die Bezahlweise? |
| Die Behandelnden haben Sie nach der Entlassung an andere Behandelnde überwiesen. |
| Konnten Sie vor der Entlassung Fragen stellen? |
| Mussten Sie während Ihres Krankenhausaufenthaltes häufig warten? |
| Die Behandelnden haben bei Schmerzen schnell reagiert und entsprechend geholfen. |
| Es fiel Ihnen leicht einen Termin zu bekommen. |
| Die Behandelnden haben Sie freundlich und empathisch behandelt. |
| Es gibt einen Behandlungsplan, der mit Ihnen besprochen wird. |
| Die Behandelnden haben Ihre Medikation aufeinander abgestimmt. |

| Dimension: Körperliche Unterstützung |
| --- |
| Die Behandelnden haben Sie in Bezug auf Ihre körperlichen Befindlichkeiten unterstützt. |
| Die Behandelnden haben Sie in Ihren körperlichen Beschwerden unterstützt, damit Sie Ihrem Alltag wieder nachgehen können. |
| Die Behandelnden haben Ihnen körperliche Unterstützung z.B. Gymnastik oder Physiotherapie angeboten. |
| Die Behandelnden haben sich bemüht um Ihnen mit Ihren körperlichen Beschwerden(z.B. Unruhe, Übelkeit) zu helfen. |
| Die Behandelnden haben sich bemüht, dass Sie sich bei der Behandlung körperlich wohlfühlen. |
| Die Behandelnden haben Sie mit Vorsicht behandelt. |
| Die Behandelnden haben Sie in Hinblick auf Ihre Ernährung informiert und unterstützt. |
| Die Essensversorgung im Krankenhaus entsprach Ihren Bedürfnissen. |
| Ihnen wurde eine Erhnährungsberater/in zur Unterstützung angeboten. |
| Die Behandelnden haben sich bemüht Ihnen Ruhe zu geben. |
| Die Behandelnden haben Sie über die zu erwartenden Schmerzen der Behandlung informiert. |
| Die Behandelnden haben sich bemüht Ihre Schmerzen zu behandeln. |
| Wie stark waren Ihre Schmerzen bei Aufnahme in das Krankenhaus / Notaufnahme? |
| Die Behandelnden haben sich bemüht herauszufinden ob Sie Schmerzen hatten. |
| Haben Sie selbst (aktiv) die Behandelden über Ihre Schmerzen informiert? |
| Wurden Ihnen Schmerzmittel während Ihrer Behandlung angeboten? |
| Die Behandelnden haben Sie auf die Entlassung vorbereitet (z.B. Gabe von ausreichender Schmerzmedikation für zuhause). |
| Die Behandelnden haben Ihre Schmerzbehandlung regelmäßig kontrolliert. |
| Die Behandelnden haben auf Ihr Verlangen nach Schmerzmedikation sofort reagiert. |
| Die Behandelnden haben sich bemüht Sie bzgl. der Organisation von administrativen Belangen aufgrund Ihrer körperlichen Einschränkungen zu unterstützen (z.B. die Beantragung eines Schwerbehindertenausweis). |
| Die Behandelnden haben Sie körperlich unterstützt (z.B. auf Toilette, zum Duschen begleitet). |
| Die Behandelnden haben auf Ihr Verlangen nach Hilfe bei der persönlichen Hygiene (Toilettengang) sofort reagiert. |
| Die Behandelnden haben mit Ihnen gemeinsam überprüft, welche Unterstützung Sie benötigen. |
| Die Behandelnden haben Ihnen erklärt, welche Unterstützungsmöglichkeiten es gibt. |

| Dimension: Patientenorientierte Merkmale |
| --- |
| Die Behandelnden haben nicht ihren Stress an Sie weitergegeben. |
| Die Behandelnden haben Ihnen das Gefühl gegeben, dass Sie im Vordergrund stehen. |
| Die Behandelnden waren positiv eingestellt. |
| Die Behandelnden waren freundlich zu Ihnen. |
| Die Behandelnden haben höflich zu Ihnen. |
| Die Behandelnden haben interessiert an Ihnen und Ihrer Gesundheit. |
| Die Behandelnden haben Mitgefühl gezeigt. |
| Die Behandelnden waren ehrlich zu Ihnen. |
| Die Behandelnden haben versucht, Sie aufzuheitern. |
| Die Behandelnden haben Sie respektvoll behandelt. |
| Die Behandelnden waren engagiert. |
| Die Behandelnden waren offen Ihnen gegenüber. |
| Die Behandelnden waren kompetent. |
| Die Behandelnden haben Ihre Emotionen/Reaktionen aufgegriffen. |
| Die Behandelnden haben darauf geachtet, ob die Behandlung zu den Lebensumständen passt. |
| Die Behandelnden haben Ihren körperlichen Beschwerden psychische Probleme unterstellt. |
| Die Behandelnden haben versucht, Sie nicht zu bestürzen. |
| Die Behandelnden haben die richtige Behandlung eingeleitet. |
| Die Behandelnden haben darauf geachtet, dass sie von anderen Behandelnden Bericht erstattet bekommen. |
| Die Behandelnden sind mit Ihrem Frust oder Sorgen umgegangen. |
| Die Behandelnden haben Sie nicht ernst genommen. |
| Wie sehr hatten Sie das Gefühl, man habe Sie in eine Schublade gesteckt, ohne Ihnen zuzuhören? |
| Die Behandelnden haben über Sie gesprochen, als ob Sie nicht da gewesen wären. |
| Die Behandelnden haben Sie gründlich untersucht. |
| Wie lange mussten Sie warten? |
| Die Behandelnden wussten über weitere Behandlungsoptionen Bescheid. |
| Die Behandelnden haben nicht nur an den Ursachen angesetzt. |
| Die Behandelnden waren in weiteren Fachbereichen ausgebildet. |
| Die Behandelnden haben Ihnen gut zugeredet. |
| Die Behandelnden haben ihre Erfahrung genutzt in der Behandlung. |
| Die Behandelnden haben sich vorgestellt, wenn Sie sie das erste Mal gesehen haben. |
| Wie kreativ waren die Behandelnden bei Ihrer Behandlung? |
| Die Behandelnden waren authentisch. |
| Die Behandlungsmethoden waren modern. |
| Die Behandelnden haben darauf geachtet, dass Sie würdevoll behandelt werden. |
| Die Behandelnden haben Ihre Meinung respektiert, auch wenn sie nicht einverstanden waren. |
| Die Behandelnden haben Sie von oben herab behandelt. |
| Die Behandelnden haben sich von Ihnen distanziert. |
| Sie konnten in einem geschützten Rahmen sprechen. |
| Die Behandelnden haben sich selbst auch Schwächen eingestehen können. |
| Die Behandelnden haben Sie wirklich behandeln und nicht nur Geld verdienen wollen. |
| Wie sehr konnten Sie zwischen medizinischem Personal (Ärztinnen und Ärzte) und weiterem Personal (Pflegekräfte oder Reinigungspersonal) unterscheiden? |
| Die Behandelnden haben Ihnen Angst gemacht durch Bemerkungen. |
| Die Behandelnden haben Ihnen etwas vorgespielt. |
| Die Behandelnden haben unnötige Daten abgefragt, um sie selbst zu nutzen. |
| Die Behandelnden haben Sie ganzheitlich betrachtet. |
| Die Behandelnden haben Sie mit Wertschätzung behandelt. |
| Die Behandelnden waren ruhig und gelassen bei der Behandlung. |
| Die Behandelnden haben sich um Sie gekümmert. |
| Die Behandelnden haben Sie dabei unterstützt eine Lösung für Ihr Gesundheitsproblem zu finden. |
| Die Behandelnden haben in der Behandlung Ihre Ressourcen berücksichtigt. |
| Die Behandelnden haben bei der Entlassung Ihren Gesundheitszustand realistisch eingeschätzt. |
| Die Behandelnden haben dafür gesorgt, dass Sie sich wohl fühlen. |
| Die Behandelnden haben Sie darauf vorbereitet was Sie nach der Behandlung brauchen. |
| Die Behandelnden haben Sie vor der Behandlung oder Untersuchung über die Kosten aufgeklärt. |
| Die Behandelnden haben darauf geachtet, dass Sie nicht zu lange warten müssen. |
| Die Behandelnden haben mit Ihnen statt über Sie gesprochen. |
| Die Behandelnden haben Fachwörter leicht verständlich ausgedrückt. |
| Die Behandelnden haben Sie nicht alleine gelassen. |
| Die Behandelnden haben Ihre eigene Expertise ibezüglich Ihrer Erkrankung abgewertet. |
| Die Behandelnden haben Sie als (einzigartigen) Menschen behandelt. |
| Die Behandelnden haben Ihnen vorher erklärt, was als nächstes gemacht wird. |
| Die Behandelnden waren arrogant. |
| Die Behandelnden haben Ihnen Ihre Gesundheitsprobleme verständlich erklärt. |
| Sie mussten darauf bestehen, dass Sie behandelt werden. |
| Sie mussten sich rechtfertigen. |
| Die Behandelnden haben sichergestellt, dass Ihre Rechte als Mensch und Patient gewahrt werden. |
| Die Behandelnden haben Sie entsprechend aktuellen, wissenschaftlichen Erkenntnissen behandelt. |
| Die Behandelnden haben Ihnen erklärt, warum Sie zu einem Facharzt überwiesen werden. |
| Behandelnde mit verschiedenen Schwerpunkten haben zusammengearbeitet um Ihre individuellen Bedürfnisse erfüllen zu können. |
| Die Behandelnden haben alle Personen fair behandelt. |
| Die Behandelnden haben Sicherheit und Zuversicht ausgestrahlt. |
| Die Behandelnden haben interdisziplinär zusammengearbeitet um Sie bestmöglich zu versorgen. |
| Die Behandelnden haben die Entscheidung getroffen, ob die Behandlung nötig ist. |
| Die Behandelnden konnten gut wahrnehmen wie akut Ihr Gesundheitsproblem ist. |
| Die Behandelnden haben Ihnen verständlich erklärt warum Sie eine Behandlung empfehlen. |
| Die Behandelnden haben schwer damit getan, Ihnen schwere Diagnosen mitzuteilen. |
| Die Behandelnden haben Sie dabei unterstützt andere Fachärzte zu kontaktieren. |
| Die Behandelnden haben Sie dabei unterstützt die Kosten von der Krankenkasse übernehmen zu lassen. |
| Die Behandelnden haben waren gut darüber informiert welche Behandlung bei Ihnen durchgeführt wird. |
| Sie konnten den Fähigkeiten der Behandelnden vertrauen. |
| Die Behandelnden konnten mit Problemen in der Behandlung umgehen. |
| Die Behandelnden waren informiert genug, um die Behandlung durchzuführen. |
| Die Behandelnden sind gut mit emotionalen Situationen umgegangen. |
| Die Behandelnden haben Ihre Prioritäten in der Behandlung berücksichtigt. |
| Die Behandelnden haben Ihnen Unterstützung bei der Bewältigung Ihrer Erkrankung angeboten. |
| Die Behandelnden haben Sie ohne Vorurteile behandelt. |
| Die Behandelnden haben sichergestellt dass die Praxis hygienisch ist. |
| Die Behandelnden haben Ihre Privatsphäre respektiert. |
| Die Behandelnden haben zu schnell gesagt, dass etwas nicht möglich ist. |
| Die Behandelnden haben Ihnen Medikamente nur gegeben, wenn es nötig und sinnvoll war. |
| Sie haben den Behandelnden vertraut. |
| Die Behandelnden haben darauf geachtet, wie Sie benötigte Medikamente vertragen. |
| Wie verärgert waren die Behandelnden, wenn Sie Fragen gestellt haben? |
| Die Behandelnden haben Dinge so einfach erklärt, dass Sie sie verstehen konnten. |
| Die Behandelnden haben Ihre Gesundheitsprobleme richtig diagnostiziert. |
| Die Behandelnden haben Sie über verschiedene Behandlungsoptionen aufgeklärt. |
| Die Behandelnden haben Ihnen beim Gespräch in die Augen gesehen. |
| Die Behandelnden sind mit Fehlern offen und ehrlich umgegangen. |
| Die Behandelnden haben Sie ausreichend gründlich untersucht. |
| Die Behandelnden haben eine ausreichende Länge Ihrer Behandlung sichergestellt. |
| Die Behandelnden haben realistische Behandlungsziele mit Ihnen besprochen. |
| Sie haben sich als Nummer gefühlt. |
| Die Behandelnden haben Ihre Beschwerden auf Ihr Gewicht geschoben. |
| Die Behandelnden haben Sie zuvorkommend behandelt. |
| Die Behandelnden haben Ihre Beschwerden auf Ihr Alter geschoben. |
| Die Behandelnden sind auf Ihre Fragen eingegangen. |
| Die Behandelnden haben einen abschätzigen Tonfall verwendet. |
| Die Behandelnden haben sich an Absprachen mit Ihnen gehalten. |
| Die Behandelnden haben Sie zu schnell behandelt, um Sie schnell zu entlassen. |
| Die Behandelnden haben bemerkt, wenn Sie etwas nicht verstanden haben. |
| Die Behandelnden waren gut ansprechbar für Sie. |
| Die Behandelnden haben Sie als gleichwertigen Gesprächspartner behandelt. |
| Die Behandelnden haben Sie wertfrei behandelt. |
| Die Behandelnden haben Sie und Ihre Situation verstanden. |
| Die Behandelnden haben sich entschuldigt, wenn sie einen Fehler gemacht haben. |
| Wie gut haben Sie sich mit den Behandelnden verstanden? |
| Die Behandelnden haben sich professionell verhalten. |
| Die Behandelnden haben respektiert, wenn Sie eine Behandlung nicht wollten. |
| Die Behandelnden wussten über Ihre Allergien Bescheid. |
| Sie mussten Beziehungen nutzen, um an Termine zu kommen. |
| Wie schnell haben Sie Termine bei ÄrztInnen erhalten? |
| Die Behandelnden haben Sie an KollegInnen mit mehr Fachexpertise vermittelt. |
| Die Behandelnden haben andere Erkrankungen bei Ihrer Behandlung beachtet. |
| Die Behandelnden haben Sie gründlich aufgeklärt. |
| Die Behandelnden haben Sie zuvorkommend behandelt. |
| Die Behandelnden haben Ihnen geglaubt, wenn Sie etwas gesagt haben. |
| Die Behandelnden haben Sie an die Hand genommen und nicht alleine gelassen. |
| Die Behandelnden haben Ihnen im Notfall geholfen. |
| Die Behandelnden haben genau zugehört und hingesehen. |
| Die Behandelnden haben sich bei einem Fehler entschuldigt. |
| Die Behandelnden haben sich vorgestellt. |
| Die Behandelnden haben darauf geachtet, dass Sie sich nicht hilflos fühlen. |
| Die Behandelnden haben Ihre Beschwerden verharmlost. |
| Die Behandelnden waren verständnisvoll. |
| Die Behandelnden haben darauf geachtet, keine Verbote auszusprechen. |
| Die Behandelnden waren geduldig. |
| Die Behandelnden haben zugegeben, wenn sie Ihnen nicht weiterhelfen konnten. |
| Die Behandelnden haben sich Zeit für Sie genommen. |
| Sie fanden die Behandelnden sympathisch. |
| Sie haben sich durch die Behandelnden willkommen gefühlt. |

| Dimension: Patientensicherheit |
| --- |
| Wie hoch war die Sicherheit während Ihrer Behandlung? |
| Sie wurden bei der Einnahme von Medikamenten durch Medikamentenplan unterstützt. |
| Die Behandelnden haben Sie überfordert. |
| Die Behandelnden haben Sie in Notfällen abgewiesen. |
| Die Behandelnden haben Sie unnötig behandelt oder untersucht. |
| Sie mussten auf eine Behandlung bestehen, damit Sie keinen Schaden davontragen. |
| Die Behandelnden haben Ihre Daten missbraucht. |
| Die Behandelnden haben dafür gesorgt, dass Sie genug Privatsphäre haben. |
| Sie konnten mit den Behandelnden in einem geschützten Rahmen sprechen. |
| Die Behandelnden haben auf Datenschutz geachtet. |
| Die Behandelnden haben Sie um Einverständnis für die Behandlung gebeten. |
| Die Behandelnden haben Sie entsprechend aktuellen wissenschaftlichen Erkenntnissen behandelt. |
| Wie vorsichtig waren die Behandelnden bei der Verordnung von Medikamenten? |
| Die Behandelnden haben Sie nicht zu schnell nach einer Behandlung entlassen. |
| Wie schnell gaben die Behandelnden auf und überwiesen Sie zu anderen Behandelnden? |
| Wie sehr wurde auf Hygiene geachtet? |
| Sie mussten eigenständig alternative Methoden ausprobieren. |
| Die Behandelnden haben nach Standards und Leitlinien gearbeitet. |
| Die Behandelnden sind gut mit Fehlern umgegangen. |
| Die Behandelnden haben Themen direkt angesprochen, anstatt sie zu umschiffen. |
| Die Behandelnden haben Ihnen die Möglichkeit gegeben, sich zu beschweren. |
| Die Behandelnden haben bei der Behandlung versucht, das Risiko so gering wie möglich zu halten. |
| Die Behandelnden haben darauf geachtet, ob Sie eine Behandlung nicht bekommen dürfen. |
| Die Behandelnden haben Ihnen erklärt, auf welche Nebenwirkungen Sie achten sollen. |
| Die Behandelnden haben Sie ans Bett gefesselt. |
| Die Behandelnden haben sich an eine Sicherheitskultur gehalten. |
| Wie sehr haben Sie sich durch andere PatientInnen belästigt gefühlt? |
| Die Behandelnden haben Ihnen erklärt, wie Sie selbstständig die Maschinen von sich entfernen können. |
| Die Behandelnden haben darauf geachtet, dass Sie keine Nebenwirkungen haben. |
| Es sind Informationen zwischen Behandelnden verloren gegangen. |
| Die Behandelnden haben Ihnen die Schuld am ausbleibenden Erfolg gegeben. |
| Sie haben eine Behandlung bekommen, die Ihnen mehr geschadet als geholfen hat. |
| Die Behandelnden haben Ihnen die Möglichkeit gegeben mitzuentscheiden oder selbst zu entscheiden, welche Behandlung durchgeführt wird |
| Die Behandelnden haben erfragt ob Sie Schmerzen haben und bei Schmerzen zügig gehandelt um Ihre Schmerzen zu senken. |
| Die Behandelnden haben jederzeit auf alle Informationen Zugriff gehabt. |
| Die Behandelnden haben Ihnen eine schriftliche Zusammenfassung darüber gegeben, welche Behandlungen bei Ihnen durchgeführt wurden oder welche Erkrankungen bei Ihnen diagnostiziert wurden. |
| Die Behandelnden wussten, wie viele Medikamente Sie erhalten haben und wann Sie neue benötigen. |
| Sie konnten selbst auf Ihre Patientenakte zugreifen. |
| Die Behandelnden haben sich vor dem Gespräch mit Ihnen über den aktuellen Stand Ihrer Gesundheit und Behandlung informiert. |
| Die Behandelnden haben Ihnen aufgeschrieben, wie die Medikamente heißen, die Ihnen gegeben wurden. |
| Wenn Sie von anderen begleitet wurden, haben diese Informationen über Sie erhalten. |
| Die Behandelnden haben Sie schriftlich über Ihre Rechte als Patient aufgeklärt. |
| Die Behandelnden haben Sie falsch behandelt. |
| Die Behandelnden haben Sie vor der Behandlung oder Untersuchung über die Kosten aufgeklärt. |
| Die Behandelnden haben auf Nebenwirkungen geachtet. |
| Die Behandelnden haben eine Behandlung fortgesetzt, obwohl Sie bei Ihnen nicht gewirkt hat. |
| Die Behandelnden haben Ihnen nur so viele Medikamente verschrieben wie Sie benötigten. |
| Die Behandelnden waren bemüht wirklich Ihr Gesundheitsproblem zu lösen. |
| Die Behandelnden haben Sie gründlich untersucht. |
| Die Behandelnden haben Ihnen verständlich erklärt, wie Sie zu Hause Ihre Behandlung fortsetzen können (z.B. Sportübungen). |
| Die Behandelnden haben Sie darüber aufgeklärt, wie Sie Fehler melden können. |
| Die Behandelnden haben eine Behandlung durchgeführt, mit der Sie nicht einverstanden waren. |
| Die Behandelnden haben darauf reagiert, wenn Sie von Nebenwirkungen berichteten. |
| Die Behandelnden haben sichergestellt, dass Ihre Patientenrechte eingehalten werden. |
| Die Behandelnden haben Hilfsmittel wie Checklisten eingesetzt, um sicherzustellen, dass alle wichtigen Schritte erfolgt sind. |
| Die Behandelnden haben Ihnen erklärt, welche Rechte Sie haben. |
| Die Behandelnden haben in einem Notfall schnell genug gehandelt. |
| Die Behandelnden haben Sie länger als notwendig behandelt. |
| Die Fragen waren zu privat. |
| Die Behandelnden haben Ihre Gesundheit ganzheitlich betrachtet. |
| Die Behandelnden haben Sie und Ihre Beschwerden ernst genommen. |
| Die Behandelnden haben Sie über verschiedene Behandlungsmethoden aufgeklärt. |
| Die Behandelnden haben sich an die Schweigepflicht gehalten. |
| Die Behandelnden haben respektiert, wenn Sie eine Behandlung abgelehnt haben. |
| Die Behandelnden haben nicht nur Symptome, sondern Ursachen behandelt. |
| Die Behandelnden haben auf Wechselwirkungen geachtet. |
| Die Behandelnden haben sich zusammengesetzt, um Ihren Fall zu besprechen. |
| Die Behandelnden haben Ihre Selbstbestimmung eingeschränkt. |
| Die Behandelnden haben akzeptiert, wenn eine Behandlungsmethode ineffektiv war. |
| Die Behandelnden haben Ihnen Schaden durch Behandlungsfehler zugefügt. |
| Sie mussten Dinge machen, die Sie nicht wollten. |

| Dimension: Persönlich angepasste Informationen |
| --- |
| Sie haben klare Informationen erhalten. |
| Die Behandelnden haben Ihnen an Sie und Ihre Bedürfnisse angepasste Informationen gegeben. |
| Die Behandelnden haben Sie ausführlich über die Behandlung aufgeklärt. |
| Die Behandelnden haben oft genug mit Ihnen gesprochen. |
| Die Behandelnden haben Sie über Behandlungsalternativen aufgeklärt. |
| Sie waren darauf angewiesen, dass Ihnen Sachen gesagt werden. |
| Sie hatten einen festen Ansprechpartner. |
| Die Behandelnden haben erklärt, wo Sie zusätzliche Informationen herbekommen. |
| Die Behandelnden haben erklärt, wo Sie Informationen durch Weiterbildung erhalten können. |
| Die Behandelnden haben erklärt, was Sie tun müssten, um Ihre Krankheit in den Griff zu bekommen. |
| Die Behandelnden haben Ihre Eigeninitiative gestärkt. |
| Wie verständnisvoll haben die Behandelnden die Informationen gegeben? |
| Die Behandelnden haben Hilfsmittel genutzt, um Ihnen Informationen zu vermitteln. |
| Wie schnell haben die Behandelnden Ihnen Informationen vermittelt? |
| Die Behandelnden haben Ihnen vermittelt, dass es wieder gut wird. |
| Die Behandelnden haben erklärt, wie Sie Ihre Behandlung zuhause fortführen können. |
| Die Behandelnden haben Ihnen die Ziele der Behandlung verständlich erklärt. |
| Wie gut konnten Sie auf den Behandlungsplan zugreifen? |
| Die Behandelnden haben gesagt, wie lange die Behandlung dauern wird. |
| Die Behandelnden haben auf Wechselwirkungen geachtet. |
| Die Behandelnden haben erklärt, welche Vor- und Nachteile die Behandlung hat. |
| Die Behandelnden haben erklärt, wie die Behandlung nach der Entlassung fortgeführt wird. |
| Alle Informationen wurden in einer Patientenakte gebündelt. |
| Wie rechtzeitig haben Sie Bescheid bekommen, dass Sie zuhause keine pflegerische Unterstützung mehr erhalten werden? |
| Die Behandelnden haben ausführlich über Testergebnisse informiert. |
| Die Behandelnden sind strukturiert vorgegangen. |
| Die Behandelnden haben Ihnen auf alternative Arten Aufklärung geboten. |
| Die Behandelnden haben mit Ihnen einen Medikationsplan erstellt. |
| Die Behandelnden haben Ihnen einen Entlassbericht mitgegeben. |
| Die Behandelnden haben Sie über Ihre Erkrankung und ihren Verlauf aufgeklärt. |
| Die Behandelnden haben Sie informiert, was Sie tun müssen, wenn Sie zuhause Probleme haben sollten. |
| Die Behandelnden haben selbst unangenehme Informationen mit Ihnen geteilt. |
| Die Behandelnden haben erklärt, wann Sie Ihren Alltagsaktivitäten wieder nachkommen können. |
| Die Behandelnden haben Ihr eigenes Wissen mitaufgenommen. |
| Die Behandelnden haben sich darüber informiert, ob Sie von Zuzahlungen befreit sind. |
| Die Behandelnden haben mit Ihnen darüber gesprochen, wie viel Sie zuzahlen müssen. |
| Die Behandelnden haben Ihnen die Möglichkeit gegeben, Ihr eigenes Wissen zu teilen. |
| Die Behandelnden haben Ihre Allergien beachtet. |
| Sie konnten Fragen stellen. |
| Die Behandelnden haben Ihre Fragen (ausführlich) beantwortet. |
| Die Behandelnden haben Ihnen Empfehlungen undTipps bezüglich Ihrer Krankheit gegeben. |
| Die Behandelnden haben Ihnen genug Informationen gegeben. |
| Die Behandelnden haben Sie als (einzelnen) Menschen behandelt. |
| Die Behandelnden haben Ihnen verständlich erklärt warum Sie eine Behandlung erhalten, wie diese wirkt und welche Vor- und Nachteile die Behandlung hat. |
| Die Behandelnden haben Ihnen verständlich erklärt warum Sie dieses Medikament bekommen, wie es wirkt, wie Sie es einnehmen sollen und wie sie mit anderen Medikamenten wechselwirken. |
| Die Behandelnden haben Ihnen verständlich erklärt warum Sie eine Untersuchung bekommen, was die Ergebnisse bedeuten und Ihnen die Ergebnisse gezeigt. |
| Die Behandelnden haben Ihnen verständlich erklärt auf welche Symptome Sie achten sollten und in wohin Sie sich in welchen Fällen wenden können. |
| Die Behandelnden haben Ihnen Ihre Möglichkeiten verständlich dargelegt. |
| Die Behandelnden haben Ihnen ausreichend Informationen gegeben damit Sie sicher eine Entscheidung zu Ihrer Behandlung treffen konnten. |
| Sie hatten Zugriff auf Ihre eigene Patientenakte. |
| Die Behandelnden haben Informationen so gegeben, dass Sie sie verstehen konnten. |
| Die Behandelnden haben Ihnen Informationen zu dem Zeitpunkt gegeben zu dem Sie sie benötigten. |
| Die Behandelnden haben Ihnen Ihre Nachsorge gut erklärt. |
| Die Behandelnden wollten wissen, was Sie an Informationen benötigen. |
| Die Behandelnden haben Ihre aktuelle Situation einfühlsam beachtet. |
| Die Behandelnden haben erklärt und aufgeschrieben, wo Sie weitere Kontakte und Unterstützung erhalten (z.B. Kontaktadressen und Telefonnummern von Selbsthilfegruppen). |
| Die Behandelnden haben Sie über den aktuellen Stand Ihrer Behandlung informiert. |
| Die Behandelnden haben sich vorgestellt und ihre Aufgabe erklärt. |
| Die Behandelnden haben Sie darüber informiert an welchem Punkt in Ihrer Behandlung Sie sind und welche Schritte folgen. |
| Mussten Sie selbst darauf bestehen, dass wichtige Untersuchungen gemacht werden (z.B. dass ein bestimmter Blutwert getestet wird)? |
| Die Behandelnden haben bei der Gabe von Medikamenten darauf geachtet, dass diese für Sie geeignet sind (Wechselwirkungen andere Medikamente, Allergien, Erkrankungen, Alter etc.). |
| Die Behandelnden haben Ihnen nur Behandlungen empfohlen die medizinisch notwendig und sinnvoll sind. |
| Die Behandelnden haben Ihre Präferenzen und Wünsche in der Behandlungsentscheidung berücksichtigt und respektiert. |
| Die Behandelnden haben Ihnen erklärt wie hoch die Wahrscheinlichkeit ist, dass die Behandlung Erfolg hat. |
| Die Behandelnden haben Ihnen erklärt, was passieren kann wenn Sie die Behandlung nicht machen. |
| Die Behandelnden haben Sie vorher darüber informiert wie unangenehm oder schmerzhaft die Behandlung oder Untersuchung sein kann. |
| Die Behandelnden haben Sie über die Prognose aufgeklärt. |
| Die Behandelnden haben erklärt, an welche Fachpersonen Sie sich wenden sollten. |
| Die Behandelnden wussten klar welche Aufgaben oder Behandlungen sie durchführen können und dürfen. |
| Die Behandelnden haben zu Beginn alle Ihre Symptome abgefragt. |
| Die Behandelnden haben Sie gefragt, wie Sie die Testergebnisse verstehen. |
| Die Behandelnden haben mit Ihnen in einem geschützten Raum gesprochen. |
| Die Behandelnden haben Ihnen verständlich erklärt warum Sie zu einem Facharzt oder Station überwiesen werden. |
| Die Behandelnden haben Ihnen erklärt wie akut oder bedrohlich Ihr Gesundheitszustand ist. |
| Die Behandelnden haben Ihnen zu Ihrer Entlassung gut verständliche, schriftliche Informationen zu Ihrer Nachsorge oder Selbstmanagement gegeben. |
| Die Behandelnden haben erklärt, weshalb Sie eine Behandlung nicht erhalten haben. |
| Die Behandelnden haben Sie fair behandelt. |
| Die Behandelnden haben Sie informiert über Angebote zur Vorsorge. |
| Die Behandelnden haben Sie nach erlebten Problemen mit den Medikamenten gefragt. |
| Sie konnten Ihre Situation schildern. |
| Die Behandelnden haben sich ausreichend Zeit für Sie genommen |
| Die Behandelnden haben konnten Ihre Fragen kompetent beantworten |
| Die Behandelnden haben Sie gründlich untersucht |
| Die Behandelnden haben verärgert auf Ihre Fragen reagiert. |
| Die Behandelnden haben reagiert, wenn Sie Bedenken geäußert haben. |
| Die Behandelnden haben Sie ausreden lassen. |
| Die Behandelnden haben abgefragt, welche Medikamente Sie einnehmen. |
| Die Behandelnden haben Sie unterstützt, wenn Sie Informationen wollten. |
| Die Behandelnden haben Ihnen erklärt, weshalb es zu Wartezeiten kommt. |
| Die Behandelnden haben versucht, Ihnen keine Angst zu machen. |
| Sie haben sich aufgefangen gefühlt durch die Behandelnden. |
| Die Behandelnden haben Sie über Nebenwirkungen aufgeklärt. |

| Dimension: Psychische Unterstützung |
| --- |
| Die Behandelnden sind auf Ihre Ängste und Sorgen eingegangen. |
| Die Behandelnden haben Sie über die Gefühle informiert, die Sie aufgrund Ihrer Erkrankung haben könnten. |
| Die Behandelnden haben Sie über die emotionalen Reaktionen, die Ihre Angehörigen (z.B. Freunde, Familie) aufgrund Ihrer Erkrankung erfahren könnten informiert. |
| Die Behandelnden haben Sie darüber informiert, wie Ihre Erkrankung sich auf Ihre Beziehungen (z.B. Familie, Freunde, andere) auswirken kann. |
| Die Behandelnden haben darüber informiert, wie Ihre Erkrankung sich auf Ihre Sexualität und Ihre Partnerschaft auswirken kann. |
| Die Behandelnden haben Sie emotional unterstützt, indem sie Ihnen Mut oder Trost zugesprochen haben. |
| Sie konnten sich Ihren Behandelnden anvertrauen. |
| Die Behandelnden haben getan was sie konnten, um Ihnen mit Ihren Beschwerden oder Unwohlsein zu helfen. |
| Die Behandelnden haben sich bemüht damit es Ihnen besser geht. |
| Die Behandelnden haben Sie bei der Bewältigung Ihrer Erkrankung unterstützt. |
| Die Behandelnden haben Sie bei Ihren Gefühlen gegenüber dem eigenen Körper unterstützt. |
| Die Behandelnden haben sich dafür interessiert, wie es Ihnen mit Ihrer Erkrankung geht. |
| Die Behandelnden haben Ihnen das Gefühl gegeben intime Anliegen mit ihnen besprechen zu können. |
| Die Behandelnden haben Ihnen psychologische Unterstützung im Zusammenhang mit Ihrer Erkrankung angeboten. |
| Die Behandelnden haben sich darüber informiert, ob Sie emotionale Unterstützung oder Hilfe in Bezug auf die Bewältigung Ihres Alltags benötigen. |

| Dimension: Shared Decision Making |
| --- |
| Sie waren darauf angewiesen, dass Ihnen Sachen gesagt wurden. |
| Die Behandelnden haben Sie in die Entscheidungsfindung einbezogen. |
| Die Behandelnden haben Ihnen Zeit gegeben, um über verschiedene Behandlungsmöglichkeiten nachzudenken. |
| Sie mussten mitüberlegen, ob eine Behandlung einen Zweck hat. |
| Haben Sie von sich aus ein Medikament abgesetzt? |
| Konnten Sie Behandlungsschritte auch ablehnen? |
| Die Behandelnden haben Ihnen mehrere Behandlungsalternativen angeboten. |
| Die Behandelnden haben Ihre Wünsche bei der Behandlung berücksichtigt. |
| Sie mussten Teile Ihrer Behandlung selbst anstoßen. |
| Die Behandelnden haben Ihnen die nötigen Informationen gegeben, um eine Entscheidung zu treffen. |
| Die Behandelnden haben zu schnell gesagt, dass eine Behandlung unmöglich ist. |
| Die Behandelnden haben Ihnen die Entscheidung bei der Behandlung überlassen. |
| Sie konnten Ihre Meinung in der Behandlung äußern. |
| Die Behandelnden haben Sie motiviert, die Verantwortung für Ihre Gesundheit zu übernehmen. |
| Die Behandelnden haben klargestellt, dass eine Entscheidung getroffen werden muss. |
| Die Behandelnden haben berücksichtigt, ob Sie überhaupt mitentscheiden möchten. |
| Die Behandelnden haben Sie bevormundet bei der Medikamenteneinnahme. |
| Die Behandelnden haben Ihnen respektvoll vermittelt, dass Sie Ihre Pläne nicht umsetzen können, ohne Risiken einzugehen. |
| Die Behandelnden haben Ihre Entscheidung respektiert, auch wenn diese anders war als die Empfehlung der Behandelnden. |
| Die Behandelnden haben Sie in die Diskussionen zur Behandlung einbezogen. |
| Die Behandelnden haben Sie einbezogen bei der Planung der Behandlung. |
| Sie konnten gemeinsam mit den Behandelnden eine Vereinbarung für das weitere Vorgehen treffen. |
| Die Behandelnden haben Sie dabei unterstützt eine Entscheidung zu treffen. |
| Die Behandelnden haben über Ihren Kopf hinweg entschieden. |
| Die Behandelnden haben mit Ihnen gemeinsam überlegt, wie die Behandlung durchgeführt wird. |
| Die Behandelnden haben Sie als Partner auf Augenhöhe behandelt. |
| Die Behandelnden haben Sie gefragt, wie sehr Sie einverstanden sind mit der Entscheidung. |
| Die Behandelnden haben Ihnen geholfen, alle Informationen zu verstehen. |
| Sie konnten selbstständig Ihre Behandlung abbrechen. |
| Die Behandelnden sind darauf eingegangen, was Sie gesagt haben. |
| Die Behandelnden haben Sie daran gehindert, mitzuentscheiden. |
| Die Behandelnden haben Sie dazu eingeladen, gemeinsam Entscheidungen zu treffen. |
| Sie konnten bestimmen, wann Sie Hilfe bekommen. |
| Die Behandelnden haben es zugelassen, dass Sie Zweifel äußern konnten. |
| Die Behandelnden haben Sie wie einen Gesprächspartner auf Augenhöhe behandelt. |
| Die Behandelnden haben Behandlungen zukommen lassen, wenn Sie danach fragten. |
| Sie konnten aktiv nachfragen. |
| Sie waren in der Lage, Übungen zuhause fortzuführen. |
| Sie mussten während Ihrer Behandlung mitdenken. |
| Sie konnten Ihr Wissen in die Behandlung einbringen. |
| Die Behandelnden haben Ihre Entscheidungen akzeptiert. |
| Sie haben die Initiative ergreifen müssen. |

| Dimension: Vertrauensvolles Miteinander |
| --- |
| Die Behandelnden haben Sie zur Weiterbehandlung an andere KollegInnen weitergeleitet. |
| Die Behandelnden haben darauf geachtet, dass Sie sich wohlfühlen. |
| Wie sehr konnten Sie den Behandelnden vertrauen? |
| Die Behandelnden gaben Ihnen das Gefühl von Sicherheit. |
| Sie kamen gut mit den Behandelnden zurecht. |
| Die Behandelnden sind Ihnen auf Augenhöhe begegnet. |
| Die Behandelnden haben Ihnen Kosten aufzwingen wollen. |
| Sie konnten die Behandelnden leicht erreichen. |
| Sie hatten Vertrauen in die Erfahrung der Behandelnden. |
| Sie konnten das Expertenwissen der Behandelnden nutzen. |
| Die Behandelnden haben Ihre Privatsphäre respektiert. |
| Sie hatten immer die gleichen Behandelnden. |
| Die Behandelnden haben Sie nicht alleine gelassen. |
| Die Behandelnden haben Sie nicht lange warten lassen. |
| Die Behandelnden haben sich Zeit gelassen. |
| Die Behandelnden haben versucht zu Ihnen Vertrauen aufzubauen. |
| Sie konnten den Behandelnden erzählen, was Sie bedrückt. |
| Die Behandelnden haben Sie gekannt. |
| Die Behandelnden haben Ihre gesamte Krankheitsgeschichte beachtet. |
| Die Behandelnden haben sich vorher über Ihre Testergebnisse informiert. |
| Die Behandelnden haben mit Ihnen in Kontakt gestanden, obwohl Sie nicht persönlich vor Ort waren. |
| Die Behandelnden haben Sie als Partner in der Behandlung gesehen. |
| Die Behandelnden haben auch über private Themen gesprochen, die nichts mit Ihrer Erkrankung zu tun haben. |
| Die Behandelnden haben Sie auch persönliche Dinge gefragt, um Sie besser kennenzulernen. |
| Die Behandelnden haben Absprachen mit Ihnen eingehalten. |
| Die Behandelnden haben sich Mühe gegeben. |
| Die Behandelnden haben gewusst, was Sie emotional beschäftigt. |
| Die Behandelnden haben Sie über Abläufe der Behandlung entsprechend informiert. |
| Die Behandelnden waren pünktlich. |
| Sie haben sich aufgehoben gefühlt. |
| Die Praxis/Klinik hat einen geeigneten Standort. |
| Sie hatten zu den Behandelnden ein besonderes Verhältnis. |
| Die Behandelnden haben dafür gesorgt, dass Sie wissen, was Sie wissen sollten. |
| Wie sehr gab es eine für Sie zuständige Person? |
| Wie häufig mussten Sie sich wiederholen? |
| Die Behandelnden haben sich über Ihre Bedürfnisse informiert. |
| Die Behandelnden haben Ihnen zugehört. |
| Die Behandelnden haben Sie verstanden. |
| Die Behandelnden haben Sie wahrgenommen. |
| Die Behandelnden haben Sie in der Behandlung unter Druck gesetzt. |

| Dimension: Zugang zur Behandlung |
| --- |
| Ihnen wurde erklärt, was Sie während Ihres Aufenthaltes auf der Station/ beim Besuch in der Ambulanz erwartet. |
| Das Anmeldeverfahren war einfach. |
| Wie lange mussten Sie von der Ankunft in der Notfallambulanz warten, bis ein Behandelnder Sie untersucht hat? |
| Wie lange mussten Sie warten, bis Sie mit einem Behandelnden sprechen konnten? |
| Wie leicht haben Sie Spezialisten für Ihr Gesundheitsproblem gefunden? |
| Falls Sie von mehreren Behandelnden betreut wurden, wurde Ihnen dies mitgeteilt. |
| Falls Sie von mehreren Behandelnden betreut wurden, konnten Sie verschiedene Behandelnde aus diesem Team ansprechen, wenn Sie Hilfe benötigten. |
| Termine wurden unerwartet abgesagt. |
| Die Behandlungen, die Sie benötigten, wurden von der Krankenkasse/Rentenversicherung übernommen. |
| Es fiel Ihnen leicht die Behandlung zu bekommen die Sie benötigten. |
| Sie konnten sich gut durch das Gesundheitssystem bewegen. |
| Die Behandelnden haben sich ausreichend Zeit für Sie genommen. |
| Die Behandelnden haben sich Zeit genommen um emotionale Probleme mit Ihnen zu besprechen. |
| Die Behandelnden haben ausreichen Zeit genommen um auch über komplizierte oder emotional schwierige Themen zu sprechen. |
| Die Behandelnden haben ausreichend Zeit genommen, damit Sie alle aktuellen Gesundheitsprobleme besprechen konnten. |
| Sie wurden fair behandelt, unabhängig von Ihrem Versicherungsstatus, finanziellen Status etc. |
| Die Behandelnden haben sich ausreichend Zeit genommen um Ihnen wichtige Fragen zu Ihrem Gesundheitsproblem zu stellen. |
| Es war leicht für Sie zu dem Ort zu kommen an dem Sie den Termin hatten. |
| Sie konnten trotz Ihrer eingeschränkten Mobilität die Ambulanz/ das Krankenhaus gut erreichen. |
| Sie konnten gut verstehen, was Sie tun sollen oder wo Sie hingehen sollen. |
| Die Termine lagen zu für Sie günstigen Zeiten. |
| Sie konnten trotz Barrieren alles gut verstehen. |
| Sie haten die Möglichkeit sich bei der Arztsuche Ratings anderer Patienten anzusehen. |
| Die Beschilderung im Krankenhaus hat Ihnen geholfen sich zurechtzufinden. |
| Die Behandelnden haben Sie dabei unterstützt einen Termin bei einem Facharzt zu kriegen. |
| Die Kosten für die Behandlung oder Untersuchung wurden im Vorhinein transparent mit Ihnen besprochen. |
| Es gab die Möglichkeit die Behandlungskosten an Ihre Finanzen anzupassen. |
| Die Behandelnden haben Sie darüber informiert, unter welchen Umständen die Krankenversicherung die Kosten der Behandlung oder Untersuchung übernimmt |
| Leistungen, die Sie selbst zahlen müssen, wurden Ihnen nicht aufgedrängt. |
| Ihnen wurde alternative Behandlungsmöglichkeiten angeboten. |
| Die Kosten für die Behandlung waren angemessen. |
| Die Behandelnden haben Sie darüber informiert welche Behandlungsalternativen von der Krankenversicherung übernommen werden. |
| Die Behandelnden haben Ihnen gut erklärt, was die nächsten Schritte in Ihrer Behandlung oder Untersuchung sind. |
| Sie hatten die Möglichkeit leicht Fachärzte in Ihrer Nähe zu finden. |
| Die Behandelnden haben Ihnen mitgeteilt, an wen Sie sich wenden können, wenn Sie Sorgen zu Ihrer Gesundheit haben. |
| Die Behandelnden haben Sie dabei unterstützt die richtigen Behandelnden für Ihr Gesundheitsproblem zu finden (z.B. gut erklärt, welcher Facharzt der richtige ist). |
| Sie haben rechtzeitig einen Termin erhalten. |
| Sie haben bei einem Akutfall schnell die nötige Behandlung bekommen. |
| Die Behandelnden sind auf Ihre Sorgen eingegangen. |
| Die Behandelnden haben konnten Ihnen eine schnelle Einschätzung Ihres Problemes geben. |
| Wie lange haben Sie im Wartezimmer gewartet |
| Sie hatten verschiedene Möglichkeiten einen Termin zu vereinbaren. |
| Sie hatten die Möglichkeit auch über Entfernung mit einem Behandelnden zu sprechen (Telefon, Chat, etc.). |
| Sie hatten die Möglichkeit sich digital beraten zu lassen. |
| Eine Pflegekraft hat zuerst kurz ihr Gesundheitsproblem erfasst bevor Sie wieder im Wartezimmer gewartet haben. |
| Die Behandelnden haben Sie als individuellen Menschen behandelt. |
| Die Behandelnden haben Ihnen Testergebniss zügig rückgemeldet. |
| Wie lange hat der gesamte Besuch gedauert? |
| Die Behandelnden haben Sie darüber informiert, wie lange Sie warten müssen. |
| Ihnen wurde mitgeteilt auf welchen Behandler Sie warten. |
| Es waren alle nötigen Geräte oder Ausrüstung für Ihre Behandlung zugänglich. |
| Die Behandelnden haben Ihnen Behandlungen verordnet, die medizinisch nicht notwendig waren. |
| Sie wurden ordentlich aufgenommen. |
| Sie wurden freundlich empfangen. |
| Die Behandelnden haben Ihnen erklärt, wo Sie die Hilfe erhalten, die Sie benötigen. |
| Wie gut konnten Sie sich zurechtfinden? |
| Wie flexibel waren die Behandelnden? |
| Sie konnten die Einwilligungserklärungen gut verstehen. |
| Die Behandelnden haben Rücksicht darauf genommen, dass Sie wenig Deutsch sprechen. |
| Die Behandelnden haben Ihnen erklärt, welche Leistungen Sie am Behandlungsort erhalten können. |
| Sie wurden darüber informiert welche Leistungen eine IGeL-Leistung und damit Mehrkosten darstellen. |
| Es gab eine Notfallsprechstunde. |
| Die Behandelnden haben Sie bedrängt, eine Behandlung selbst zu bezahlen. |
| Die Behandelnden haben Sie ernst genommen. |
| Es gab ausreichend Sitzmöglichkeiten im Wartebereich. |
| Die Behandelnden haben Sie kompetent behandelt. |
| Ihr Daten wurden vertraulich behandelt. |
| Sie haben auch außerhalb der Öffnungszeiten Hilfe erhalten. |
| Überweisungen zwischen Ärzten oder Krankenhäusern liefen reibungslos. |
| Die Behandelnden waren kompetent. |
| Es gab ausreichend Parkplätze. |
| Sie konnten Formulare, die Ihnen gegeben wurden, gut verstehen. |
| Die Behandelnden haben sich ausreichend Zeit für die Untersuchung genommen. |
| Die Behandelnden haben Ihnen aufmerksam zugehört. |
| Die Behandelnden waren engagiert. |

| Dimension: Zusammenarbeit Behandler |
| --- |
| Sie konnten sich auf die Hilfe aller Behandelnden im Team verlassen. |
| Sie waren den Behandelnden des Teams bekannt. |
| Die Behandelnden haben Ihnen andere (Fach-)Ärzte empfohlen und Sie dorthin vermittelt. |
| Die Behandelnden des Teams wussten klar wer für welche Aufgabe zuständig ist. |
| Die Behandelnden des Teams haben sich gut abgesprochen. |
| Wie oft mussten Sie Informationen wiederholen die Sie bereits anderen Behandlern mitgeteilt haben? |
| Verschiedene Behandler haben Ihnen widersprüchliche Information gegeben. |
| Sie hatten einen festen Ansprechpartner, der darauf geachtet hat, dass Sie alle Informationen erhalten. |
| Nach der Entlassung aus dem Krankenhaus wusste Ihr Hausarzt über die Behandlung Bescheid (z.B. Änderung in Medikation etc.). |
| Sie hatten einen festen Ansprechpartner der Ihre Behandlung koordiniert hat. |
| Alle beteiligten Behandelnden hatten Zugriff auf Ihre Patientenakte. |
| Die Behandelnden waren gut über Sie und Ihre Gesundheit informiert. |
| Zwischen Behandelnden abgesprochene Termine wurden eingehalten. |
| Die Behandelnden haben Ihnen erklärt warum Sie andere Fachärzte besuchen sollen. |
| Die Behandelnden des Teams arbeiteten gut zusammen. |
| Wenn die Expertise der Behandelnden nicht ausreichend war, haben sie sichergestellt, dass ein anderer Behandelnder mit der Expertise Sie versorgt. |
| Die Behandelnden waren gut mit anderen Fachbereichen vernetzt und konnten Sie dorthin weitervermitteln. |
| Durch die Zusammenarbeit verschiedener Fachbereiche wurde Ihre gesamte Gesundheit betrachtet (ganzheitlicher Ansatz), |
| Die Behandelnden gehen respektvoll und wertschätzend miteinander um. |
| Wie oft haben Sie bei einem Termin Ihren Hausarzt persönlich gesehen? |
| Sie hatten einen festen Ansprechpartner, der verfügbar war um Ihnen Fragen zu beantworten. |
| Verschiedene Behandelnde haben eine gute Vorstellung von dem Beruf der anderen Behandelnden. |
| Die Praxis/Station war gut organisiert. |
| Es wurde auf Hierarchie bei der Teamarbeit verzichtet. |
| Die Behandelnden haben Informationen über Sie an Ihre Ansprechperson weitergeleitet. |
| Die Behandelnden wussten über die aktuelle Entwicklung Ihrer Behandlung Bescheid. |
| Die Behandelnden haben sich regelmäßig besprochen, um Ihre Situation gut einzuschätzen. |
| Die Behandelnden haben mit Behandelnden anderer Disziplinen zusammengearbeitet. |
| Die Behandelnden haben sich im Team respektvoll verhalten. |
| Andere Meinungen im Team werden zugelassen. |
| Das Team von Behandelnden hat sich gut organisiert. |
| Auf der Station/in der Praxis gab es eine angenehme Stimmung. |
| Es gibt eine Supervision/Intervision für das Behandlungsteam. |
| Die Behandelnden des Teams haben gleiche Begriffe verwendet. |
| Die Leitung fördert die Zusammenarbeit im Team. |
| Die Behandelnden im Team arbeiten vertrauensvoll miteinander. |
| Sie fühlten sich in sicheren Händen. |
| Die Behandelnden des Teams verfolgten gemeinsame Ziele. |
